# Supplementary material for: A multi-resolution systematically improvable quantum embedding scheme for large-scale surface chemistry calculations
Source: Nat Commun. 2025 Oct 21;16:9297. doi: 10.1038/s41467-025-64374-2 (PMC12540692; doi:10.1038/s41467-025-64374-2)
Supplement: Supplementary file 1 — Supplementary Information [file 41467_2025_64374_MOESM1_ESM.pdf]

# Supporting Information: Advancing Surface Chemistry with Large-Scale Ab-Initio Quantum Many-Body Simulations

Zigeng Huang[1*], Zhen Guo[1], Changsu Cao[1], Hung Q. Pham[2], Xuelan Wen[1], George H. Booth[3*], Ji Chen[4,5*], Dingshun Lv[1*]

[1]ByteDance Research, Fangheng Fashion Center, No. 27, North 3rd Ring West Road, Haidian District, Beijing 100098, People's Republic of China.
[2]ByteDance Research, San Jose, CA 95110, United States.
[3]Department of Physics, King's College London, Strand, London WC2R 2LS, United Kingdom
[4]School of Physics, Peking University, Beijing 100871, People's Republic of China.
[5]Interdisciplinary Institute of Light-Element Quantum Materials, Frontiers Science Center for Nano-Optoelectronics, Peking University, Beijing 100871, People's Republic of China.

*Corresponding author(s). E-mail(s): huangzigeng@bytedance.com; george.booth@kcl.ac.uk; ji.chen@pku.edu.cn; lvdingshun@bytedance.com;

## Contents

**S1 Methods** 2
S1.1 Systematically Improvable Quantum Embedding Workflow . . . 2
S1.2 Partition wavefunction density matrix scheme . . . 4
S1.2.1 Global 1-RDM . . . 5
S1.2.2 In-cluster 2-RDM . . . 8
S1.2.3 Energy Calculation in PWF-DM . . . 9
S1.3 Perturbative (T) in SIE . . . 9
S1.3.1 The In-situ Form . . . 9
S1.3.2 The Ex-situ Form . . . 11
S1.4 Complete Basis Set Extrapolation . . . 12
S1.5 The Choice of BNO Threshold . . . 13
S1.6 Linear Scaling for SIE+CCSD(T) . . . 13

**S2 GPU-Accelerated High-Performance Package** 15
S2.1 SIE framework . . . 15
S2.2 Electron repulsion integral generation . . . 15
S2.3 High-level solver: MP2 . . . 16
S2.4 High-level solver: CCSD/CCSD(T) . . . 16
S2.5 PWF-DM . . . 16

**S3 Water molecule on graphene** 17
S3.1 Outline for Achieving OBC-PBC "Handshake" . . . 17
S3.2 Structures . . . 18
S3.3 Partition Strategy . . . 18
S3.4 Graphene-Water Distance Optimization . . . 19
S3.5 Graphene-Water Distance Optimization under different water orientation . . . 20

S3.6 Interacting Energy Calculation with SIE+CCSD(T) . . . 21
S3.7 Bath truncation error correction . . . 21
S3.8 Bulk Limit Extrapolation . . . 24
S3.9 DFT Study and Geometry Relaxation . . . 25
S3.10 $H_2O$@Graphene adsorption energy . . . 26
S3.11 Interacting energy calculations under different water orientation . . . 27
S3.12 Electron density rearrangement . . . 27
S3.13 Adsorption-Induced Dipole Moment . . . 28
S3.14 Weak Interaction Analysis . . . 29

**S4 Carbonaceous molecules on various surfaces** **30**
S4.1 CO@MgO(001) . . . 30
S4.2 Organic Molecules@Coronene . . . 32
S4.3 CO/$CO_2$@CPO-27-Mg Metal-Organic Framework . . . 34

**S5 Computational details for SIE calculations** **35**

# S1 Methods

## S1.1 Systematically Improvable Quantum Embedding Workflow

The main workflow of systematically improvable quantum embedding (SIE) follows Ref. [1]. SIE starts from the low-level processing. Hatree-Fock (HF) method is used as the low-level solver throughout this work. With the HF result, the low-level one-body reduced density matrix (1-RDM) and the localized atomic orbitals are obtained. The intrinsic atomic orbital (IAO) localization method is used to localize the molecular orbitals obtained from HF. The fragment is formed by IAOs. By using low-level 1-RDM in IAO, the bath that corresponds to **x**-th fragment is constructed by Schmidt decomposition. Here, we perform the Schmidt decomposition by diagonalizing the environment part of the low-level 1-RDM that does not include the orbitals of fragment **x**. To do this, the $\boldsymbol{\gamma}^{\mathrm{HF}}$ under localized orbital basis (LO) should be reorganized by moving each row and column in the form of

$$\boldsymbol{\gamma}^{\mathrm{HF}} = \begin{bmatrix} \boldsymbol{\gamma}^{\mathrm{frag}} & \boldsymbol{\gamma}^{\mathrm{inter}} \\ \boldsymbol{\gamma}^{\mathrm{inter}\dagger} & \boldsymbol{\gamma}^{\mathrm{env}} \end{bmatrix}. \tag{S1}$$

The bath orbitals are obtained by diagonalizing $\boldsymbol{\gamma}^{\mathrm{env}}$, like

$$\boldsymbol{\gamma}^{\mathrm{env}} = \boldsymbol{C}^{\mathrm{LO}\to\mathrm{env}} \boldsymbol{\Lambda}^{\mathrm{env}} \boldsymbol{C}^{\mathrm{LO}\to\mathrm{env}\dagger}, \tag{S2}$$

where the diagonal eigenvalue matrix $\boldsymbol{\Lambda}^{\mathrm{env}}$ represents the occupation numbers of the environment orbitals. For a closed-shell system, orbitals with occupation numbers between 0 and 2 that exhibit entanglement with fragment orbitals at the HF level are termed bath orbitals. Conversely, the fully occupied or unoccupied orbitals signify unentangled environment orbitals. The matrix $\boldsymbol{C}^{\mathrm{LO}\to\mathrm{env}}$ represents the transformation coefficients from LO to these environment orbitals. By incorporating fragment orbitals, we derive a new set of orbitals, termed embedding orbitals (EO), with the coefficient transformation from LO to EO represented as

$$\boldsymbol{C}^{\mathrm{LO}\to\mathrm{EO}} = \begin{bmatrix} \boldsymbol{I} & 0 \\ 0 & \boldsymbol{C}^{\mathrm{LO}\to\mathrm{env}} \end{bmatrix}. \tag{S3}$$

EO is composed of fragment, bath, fully occupied and virtual unentangled environment orbitals. Therefore, $\boldsymbol{C}^{\mathrm{LO}\to\mathrm{EO}}$ is split into 4 parts in column dimension, like

$$\boldsymbol{C}^{\mathrm{LO}\to\mathrm{EO}} = \boldsymbol{C}^{\mathrm{LO}\to\mathrm{EO(frag)}} \oplus \boldsymbol{C}^{\mathrm{LO}\to\mathrm{EO(bath)}} \oplus \boldsymbol{C}^{\mathrm{LO}\to\mathrm{EO(occ)}} \oplus \boldsymbol{C}^{\mathrm{LO}\to\mathrm{EO(vir)}}, \tag{S4}$$

where the

$$\boldsymbol{C}^{\mathrm{LO}\to\mathrm{EO(frag)}} = [\boldsymbol{I}, 0]^{\top}, \tag{S5}$$

$$\boldsymbol{C}^{\mathrm{LO}\to\mathrm{EO(bath)}} = [0, \boldsymbol{C}^{\mathrm{LO}\to\mathrm{env(bath)}}]^{\top}, \tag{S6}$$

$$\boldsymbol{C}^{\mathrm{LO}\to\mathrm{EO(occ)}} = [0, \boldsymbol{C}^{\mathrm{LO}\to\mathrm{env(occ)}}]^{\top}, \tag{S7}$$

$$\boldsymbol{C}^{\mathrm{LO}\rightarrow\mathrm{EO(vir)}} = [0, \boldsymbol{C}^{\mathrm{LO}\rightarrow\mathrm{env(vir)}}]^{\top}. \tag{S8}$$

At the mean-field level, unentangled environment orbitals are indistinguishable from the fragment+bath subspace (FBS), as they could be either fully occupied or fully empty. However, typically, the number of bath orbitals is commensurate with that of the fragment, which makes the FBS scale insufficient for capturing significant correlations that may contribute from these unentangled environment orbitals. To relieve this problem, going beyond mean-field approaches, like correlated methods, is necessary to capture the entanglement between the unentangled environment and the FBS, comprised of the fragment and bath orbitals. Within the SIE framework, this entanglement is assessed through MP2 within two separate subspaces: one formed by the occupied orbitals of the environment and the virtual orbitals of the FBS, and another formed by the virtual orbitals of the environment and the occupied orbitals of the FBS. As MP2 calculations are confined to these subspaces, and considering the number of orbitals in the FBS is small enough to be considered constant, the MP2 costs are confined to $O(N^3)$. We then construct Fock matrix $\boldsymbol{F}^{\mathrm{FBS}}$ by projecting the entire system's Fock matrix into the FBS space, following the equation

$$\boldsymbol{F}^{\mathrm{FBS}} = \boldsymbol{C}^{\mathrm{LO}\rightarrow\mathrm{EO(FBS)}\dagger}\boldsymbol{F}\boldsymbol{C}^{\mathrm{LO}\rightarrow\mathrm{EO(FBS)}}, \tag{S9}$$

where $\boldsymbol{F}$ is the full system Fock matrix in LO basis, and $\boldsymbol{C}^{\mathrm{LO}\rightarrow\mathrm{EO(FBS)}}$ is the transformation matrix from LO to FBS, defined as

$$\boldsymbol{C}^{\mathrm{LO}\rightarrow\mathrm{EO(FBS)}} = [\boldsymbol{C}^{\mathrm{LO}\rightarrow\mathrm{EO(frag)}}, \boldsymbol{C}^{\mathrm{LO}\rightarrow\mathrm{EO(bath)}}]. \tag{S10}$$

Then, by diagonalizing the $\boldsymbol{F}^{\mathrm{FBS}}$, the occupied and virtual orbitals in FBS could be described as a transformation matrix from EO to these orbitals, like

$$\boldsymbol{F}^{\mathrm{FBS}} = \boldsymbol{C}^{\mathrm{EO(FBS)}\rightarrow\mathrm{FBS}}\boldsymbol{\varepsilon}^{\mathrm{FBS}}\boldsymbol{C}^{\mathrm{EO(FBS)}\rightarrow\mathrm{FBS}\dagger}. \tag{S11}$$

The $\boldsymbol{C}^{\mathrm{EO(FBS)}\rightarrow\mathrm{FBS}}$ denotes the transformation matrix. The $\boldsymbol{\varepsilon}^{\mathrm{FBS}}$ here denotes the molecular orbitals energies in FBS. And the transformation matrix from LO to FBS molecular orbitals is obtained as

$$\boldsymbol{C}^{\mathrm{LO}\rightarrow\mathrm{FBS}} = \boldsymbol{C}^{\mathrm{LO}\rightarrow\mathrm{EO(FBS)}}\boldsymbol{C}^{\mathrm{EO(FBS)}\rightarrow\mathrm{FBS}}. \tag{S12}$$

The transformation matrix from LO to the subspace composed of unentangled environment occupied orbital (eo) and FBS virtual orbitals (sv) denotes $\boldsymbol{C}^{\mathrm{LO}\rightarrow\mathrm{sub(eo,sv)}}$ as

$$\boldsymbol{C}^{\mathrm{LO}\rightarrow\mathrm{sub(eo,sv)}} = [\boldsymbol{C}^{\mathrm{LO}\rightarrow\mathrm{EO(occ)}}, \boldsymbol{C}^{\mathrm{LO}\rightarrow\mathrm{FBS(vir)}}], \tag{S13}$$

and the transformation matrix from LO to the subspace composed of FBS occupied orbital (so) and unentangled environment virtual orbitals (ev) denotes $\boldsymbol{C}^{\mathrm{LO}\rightarrow\mathrm{sub(so,ev)}}$ as

$$\boldsymbol{C}^{\mathrm{LO}\rightarrow\mathrm{sub(so,ev)}} = [\boldsymbol{C}^{\mathrm{LO}\rightarrow\mathrm{FBS(occ)}}, \boldsymbol{C}^{\mathrm{LO}\rightarrow\mathrm{EO(vir)}}]. \tag{S14}$$

Using these transformation matrices, the Hamiltonian can be truncated from the full system under the LO basis into the subspace, allowing for the performance of MP2 calculations within this subspace. The central double excitation $t_{ij}^{ab}$ in MP2 is denoted as

$$t_{ij}^{ab} = -(ia|jb)/D_{ij}^{ab}, \tag{S15}$$

$$D_{ij}^{ab} = \epsilon_a + \epsilon_b - \epsilon_i - \epsilon_j \tag{S16}$$

where the $\epsilon_i(\epsilon_j)$ and $\epsilon_a(\epsilon_b)$ are the occupied orbital energy, the virtual orbital energy, respectively, and $ijkl$ $(abcd)$ denote for the occupied(virtual) orbital. The 1-RDM could be obtained by

$$\gamma_{ab} = 2\sum_{ij}\sum_{c} t_{ij}^{ac}(2t_{ij}^{bc} - t_{ij}^{cb}), \tag{S17}$$

$$\gamma_{ij} = 2\delta_{ij} - 2\sum_{k}\sum_{ab} t_{ik}^{ab}(2t_{jk}^{ab} - t_{jk}^{ba}), \tag{S18}$$

where the $\gamma_{ab}(\gamma_{ij})$ denotes the virtual(occupied) part of 1-RDM. The inter part between the virtual and occupied part in 1-RDM is 0 for MP2. Diagonalizing the 1-RDM yields the bath natural orbitals (BNOs) as eigenvectors, where the eigenvalues correspond to the population on the BNOs. The

variation in population (comparing MP2 with HF) shows the level of entanglement between the BNOs and the FBS, allowing for the ranking of BNOs. Therefore, we focus only on the part of the subspace attributed to the environment. Specifically, in the subspace composed of the occupied orbital in the unentangled environment and virtual orbitals in FBS, all occupied orbitals come from the environment. Therefore, we only need to consider the $\gamma_{ij}$, similarly for the virtual orbitals from the unentangled environment. The diagonalization process is similar to the Eq. S2 and not repeated here.

The complete set of BNOs is derived from both the occupied and virtual orbitals within the unentangled environment. The corresponding transformation matrix from LO to BNO could be obtained by

$$\boldsymbol{C}^{\mathrm{LO}\to\mathrm{BNO}} = [\boldsymbol{C}^{\mathrm{LO}\to\mathrm{EO(occ)}}\boldsymbol{C}^{\mathrm{EO(occ)}\to\mathrm{BNO(occ)}}, \boldsymbol{C}^{\mathrm{LO}\to\mathrm{EO(vir)}}\boldsymbol{C}^{\mathrm{EO(vir)}\to\mathrm{BNO(vir)}}]. \tag{S19}$$

The BNO threshold, $\eta$, is utilized to truncate the BNOs. Orbitals with population variation surpassing $\eta$ are deemed significant at the MP2 level due to their correlation with the FBS and are incorporated into the FBS to form the SIE cluster. Therefore, the transformation matrix $\boldsymbol{C}^{\mathrm{LO}\to\mathrm{clu}}$ from LO to the SIE cluster is defined by

$$\boldsymbol{C}^{\mathrm{LO}\to\mathrm{clu}} = [\boldsymbol{C}^{\mathrm{LO}\to\mathrm{EO(FBS)}}, \boldsymbol{C}^{\mathrm{LO}\to\mathrm{BNO}(\eta)}], \tag{S20}$$

where the $\boldsymbol{C}^{\mathrm{LO}\to\mathrm{BNO}(\eta)}$ get the BNOs whose population variation is larger than $\eta$. After obtaining the cluster's orbitals, the cluster Hamiltonian can be truncated from the full system, allowing for direct computation using a high-level solver. Note that the addition of bath and BNO will normally introduce extra electrons, which must be considered in actual calculations. With this, the main part of the SIE workflow is complete.

The MP2-ranked BNOs are identified as a source for the systematic improvability in the SIE framework. Orbitals within the unentangled environment exhibit a significant correlation with the FBS at the MP2 level. Therefore, incorporating more of these orbitals into the cluster allows for a better representation of the entanglement between FBS andenvironment. This improved representation leads to more accurate final results [1, 2].

The origin of BNOs is not strictly limited to the unentangled environment. Ref. [1] mentioned the direct usage of the full system's molecular orbitals to construct BNOs. In this context, before BNO construction, the MP2-derived 1-RDM is truncated to the unentangled environment space to ensure there is no overlap with the FBS. The outcomes sourced from these two origins of BNOs do not substantially diverge because the environment is considerably large compared to the FBS.

## S1.2 Partition wavefunction density matrix scheme

Nusspickel et al. demonstrated four distinct strategies for recovering total-energy expectation values in quantum embedding theory [2]:

- Democratic partitioning of density matrix (DPDM). This is the default merging approach adopted in DMET. Each cluster's reduced density matrix (RDM) are first projected into the corresponding fragment. The all fragment-projected RDMs are then merged along the fragment index—without further modification—to yield the global RDMs of the full system.

- Democratic partitioning of the cumulant (DPDM-C). DPDM-C refines DPDM by retaining the DPDM construction for the global one-body RDM (1-RDM) while building the global two-body RDM (2-RDM) from the mean-field 2-RDM augmented by the cluster cumulants obtained with the high-level quantum many-body solver. Because the cumulant expansion couples 1-RDMs between different clusters (cf. Eq. 13, 14, 16 in Ref. [2]), cross-cluster information is implicitly incorporated, leading to a systematic accuracy improvement over plain DPDM.

- Partition wavefunction in exponential form (PWF-EF). Within a configuration-interaction (CI) perspective, the cluster wave function can be expressed in terms of excitation amplitudes. PWF-EF projects these amplitudes into the fragment space and evaluates and sums over each fragment's contribution to the correlation energy directly from this fragment-projected amplitudes, finally adding the mean-field energy to obtain the total energy.

- Partition wave-function density matrix (PWF-DM). This is the protocol adopted in this paper SIE workflow. Fragment-projected amplitudes are first used to reconstruct the RDMs, after which the total energy is computed in the usual RDM formalism. Relative to PWF-EF, which accounts

only for in-cluster interaction, PWF-DM includes contractions between amplitudes belonging to different clusters when building the global 1-RDM, thereby including explicit cross-cluster information. Consequently, PWF-DM is strictly more accurate than PWF-EF.

Although DPDM-C likewise incorporates cross-cluster information and can therefore capture part of the long-range correlation, it inherits the fundamental drawback of DPDM, that is the global RDM is obtained by a merging of fragment-projected RDM and is not $N$-representable. In other words, it is hard to find a wave function whose exact RDMs coincide with the DPDM-C construction. As a consequence, some basic quantum numbers, such as the total electron number, can be not preserved. Therefore, this non-N-representable expression form will introduce uncertainty to result. And, in practical calculation, one must supplement DPDM-C with iterative fitting procedures (e.g. chemical potential fitting) to preserve good quantum number. These extra fitting layer substantially increases the computational overhead. And the fitting may suffer from convergence instabilities, especially when a multi-layer fitting is imposed to constraint several quantum number simultaneously.

By contrast, the PWF series of approaches constructs the output directly from the partitioned cluster wave functions and is therefore inherently $N$-representable. No ad-hoc fitting is needed, and most good quantum numbers are respected by PWF construction. Based on this, PWF-DM strictly more accurate than PWF-EF as discussed, while the additional computational cost is modest and never exceeds the overall scaling of the SIE framework. For these reasons we adopt PWF-DM as the default energy-evaluation scheme in this work.

PWF-DM is constructed from all SIE clusters' CCSD or MP2 solutions. Here we use the restricted CCSD high-level solver as an example to get the full system RDM. The RDM at the CCSD(T) level can be achieved by incorporating methodologies, the ex-situ form of perturbative (T), which is discussed in Sec. S1.3.2 but further refinement for this form is necessary as discussed. When utilizing MP2 as the high-level solver, it is important to note that MP2 has no single excitation amplitude. Thus, in calculating PWF-DM for MP2, terms related to single excitation amplitude can be simply omitted.

In this paper, we follow the original recommendations for calculating RDMs using PWF-DM. For one-body RDM(1-RDM), we construct a global 1-RDM for the entire system. This approach introduces contractions between different cluster solutions, effectively incorporating cluster interactions and thereby enhancing accuracy. Due to computational limitations, we employ an in-cluster method for calculating the two-body RDM, meaning that the calculation is performed within each SIE cluster without inter-cluster computations. Although the in-cluster 2-RDM lacks explicit cluster interactions, a certain error cancellation mechanism inherent to this approach ensures that it outperforms a global 2-RDM. For further details, please refer to the original article [2].

### S1.2.1 Global 1-RDM

The calculation of the PWF-DM necessitates the solution of the CCSD for each cluster. Specifically, this includes $t_i^a$ and $t_{ij}^{ab}$, which are obtained from solving the CCSD equations. Additionally, it requires $\lambda_a^i$ and $\lambda_{ab}^{ij}$, which are derived from solving the CCSD $\Lambda$-equation Here, we adhere to the convention that $ijklm(abcde)$ denotes the occupied(virtual) orbital index within the SIE cluster, $f$ denotes the fragment orbital index in the LO basis, $\mathbf{x}$ and $\mathbf{y}$ denote the indices of a different cluster. The $IJKLM(ABCDE)$ denotes the occupied(virtual) orbitals index of the full system. For the 1-RDM, the original paper [2] suggests a global construction. This approach ensures the inclusion of cluster-cluster interactions. For the normal step in building the occupied part in CCSD 1-RDM [3], it would proceed as follows

$$\gamma_{IJ} = 2\delta_{IJ} - P_{IJ}\left(\sum_{A} t_I^A \lambda_A^J + \sum_{KAB} \theta_{IK}^{AB} \lambda_{AB}^{JK}\right), \tag{S21}$$

where $\theta$ defines as

$$\theta_{IJ}^{AB} = 2t_{IJ}^{AB} - t_{IJ}^{BA}. \tag{S22}$$

$P_{IJ}$ denotes the summation operation with indices $I$ and $J$ permutation like

$$P_{IJ}(X) = X_{IJ} + X_{JI}. \tag{S23}$$

The $t_I^A$ and $\lambda_A^J$ are the single excitation amplitudes for full system, which are formed by using the amplitudes in each cluster, like

$$t_I^A = \sum_{\mathbf{x}} \sum_{f_{\mathbf{x}} a_{\mathbf{x}}} t_{f_{\mathbf{x}}}^{a_{\mathbf{x}}} C_{f_{\mathbf{x}} I} C_{a_{\mathbf{x}} A}, \tag{S24}$$

$$\lambda_A^I = \sum_{\mathbf{x}} \sum_{f_\mathbf{x} a_\mathbf{x}} \lambda_{a_\mathbf{x}}^{f_\mathbf{x}} C_{f_\mathbf{x} I} C_{a_\mathbf{x} A}, \tag{S25}$$

where the $C_{f_\mathbf{x} I}$ ($C_{a_\mathbf{x} A}$) is the transformation matrix from the full system occupied (virtual) orbitals to fragment (virtual) orbitals of the cluster $\mathbf{x}$. Those transformation matrices could be obtained by contracting the inter basis. For example, the transformation matrix from full system occupied orbitals to the cluster $\mathbf{x}$ fragment orbitals could be obtained like

$$\boldsymbol{C}^{\text{EO(frag)}\to\text{MO(occ)}} = \boldsymbol{C}_\mathbf{x}^{\text{LO}\to\text{EO(frag)}\dagger} \boldsymbol{C}^{\text{LO}\to\text{MO(occ)}}, \tag{S26}$$

where the $\boldsymbol{C}_\mathbf{x}^{\text{LO}\to\text{EO(frag)}}$ is the transformation matrix from localized orbitals to the cluster $\mathbf{x}$ fragment orbitals and the $\boldsymbol{C}^{\text{LO}\to\text{MO(occ)}}$ is the transformation matrix from localized orbitals to the full system occupied orbitals. The $t_{f_\mathbf{x}}^{a_\mathbf{x}}$ and $\lambda_{a_\mathbf{x}}^{f_\mathbf{x}}$ are obtained from truncating the single excitation amplitudes of SIE cluster, like

$$t_{f_\mathbf{x}}^{a_\mathbf{x}} = \sum_{i_\mathbf{x}} t_{i_\mathbf{x}}^{a_\mathbf{x}} C_{i_\mathbf{x} f_\mathbf{x}}, \tag{S27}$$

$$\lambda_{a_\mathbf{x}}^{f_\mathbf{x}} = \sum_{i_\mathbf{x}} \lambda_{a_\mathbf{x}}^{i_\mathbf{x}} C_{i_\mathbf{x} f_\mathbf{x}}, \tag{S28}$$

where $C_{i_\mathbf{x} f_\mathbf{x}}$ is the transformation matrix in cluster $\mathbf{x}$ from the occupied orbitals to the fragment orbitals. Therefore, the first part in occupied-occupied 1-RDM denotes

$$t_I^A \lambda_A^J = \left( \sum_{\mathbf{x}} \sum_{f_\mathbf{x} i_\mathbf{x} a_\mathbf{x}} t_{i_\mathbf{x}}^{a_\mathbf{x}} C_{i_\mathbf{x} f_\mathbf{x}} C_{f_\mathbf{x} I} C_{a_\mathbf{x} A} \right) \left( \sum_{\mathbf{y}} \sum_{f_\mathbf{y} i_\mathbf{y} a_\mathbf{y}} \lambda_{a_\mathbf{y}}^{i_\mathbf{y}} C_{i_\mathbf{y} f_\mathbf{y}} C_{f_\mathbf{y} I} C_{a_\mathbf{y} A} \right). \tag{S29}$$

The memory complexity scales as $\mathcal{O}(N^2)$ for single excitation amplitudes, posing no significant consumption on memory resources, which allows for the computation of complete $t_I^A$ and $\lambda_A^J$ before proceeding with the calculations of global 1-RDM. However, for double excitation amplitudes, the size escalates to $\mathcal{O}(N^4)$ for the full system, presenting a potential storage challenge. Hence, when computing the second part of the occupied-occupied 1-RDM, $\sum_{KAB} \theta_{IK}^{AB} \lambda_{AB}^{JK}$, simplifications are necessary to avoid excessive memory consumption.

The same process in Eq. S24 and Eq. S25 could be performed on $\theta_{IJ}^{AB}$ and $\lambda_{AB}^{IJ}$, like

$$\theta_{IJ}^{AB} = \sum_{\mathbf{x}} \sum_{f_\mathbf{x} i_\mathbf{x} j_\mathbf{x} a_\mathbf{x} b_\mathbf{x}} \theta_{i_\mathbf{x} j_\mathbf{x}}^{a_\mathbf{x} b_\mathbf{x}} C_{i_\mathbf{x} f_\mathbf{x}} C_{I f_\mathbf{x}} C_{J j_\mathbf{x}} C_{A a_\mathbf{x}} C_{B b_\mathbf{x}}, \tag{S30}$$

$$\lambda_{AB}^{IJ} = \sum_{\mathbf{x}} \sum_{f_\mathbf{x} i_\mathbf{x} j_\mathbf{x} a_\mathbf{x} b_\mathbf{x}} \lambda_{a_\mathbf{x} b_\mathbf{x}}^{i_\mathbf{x} j_\mathbf{x}} C_{i_\mathbf{x} f_\mathbf{x}} C_{I f_\mathbf{x}} C_{J j_\mathbf{x}} C_{A a_\mathbf{x}} C_{B b_\mathbf{x}}. \tag{S31}$$

Therefore, the $\sum_{KAB} \theta_{IK}^{AB} \lambda_{AB}^{JK}$ could be rewritten as

$$\begin{aligned} \sum_{KAB} \theta_{IK}^{AB} \lambda_{AB}^{JK} = \sum_{KAB} & \left( \sum_{\mathbf{x}} \sum_{f_\mathbf{x} i_\mathbf{x} j_\mathbf{x} a_\mathbf{x} b_\mathbf{x}} \theta_{i_\mathbf{x} j_\mathbf{x}}^{a_\mathbf{x} b_\mathbf{x}} C_{i_\mathbf{x} f_\mathbf{x}} C_{I f_\mathbf{x}} C_{K j_\mathbf{x}} C_{A a_\mathbf{x}} C_{B b_\mathbf{x}} \right) \\ & \left( \sum_{\mathbf{y}} \sum_{f_\mathbf{y} i_\mathbf{y} j_\mathbf{y} a_\mathbf{y} b_\mathbf{y}} \lambda_{a_\mathbf{y} b_\mathbf{y}}^{i_\mathbf{y} j_\mathbf{y}} C_{i_\mathbf{y} f_\mathbf{y}} C_{J f_\mathbf{y}} C_{K j_\mathbf{y}} C_{A a_\mathbf{y}} C_{B b_\mathbf{y}} \right) \\ = & \sum_{\mathbf{xy}} \sum_{f_\mathbf{x} f_\mathbf{y}} \sum_{j_\mathbf{x} j_\mathbf{y}} \sum_{a_\mathbf{x} a_\mathbf{y}} \sum_{b_\mathbf{x} b_\mathbf{y}} \theta_{f_\mathbf{x} j_\mathbf{x}}^{a_\mathbf{x} b_\mathbf{x}} \lambda_{a_\mathbf{y} b_\mathbf{y}}^{f_\mathbf{y} j_\mathbf{y}} C_{j_\mathbf{x} j_\mathbf{y}} C_{a_\mathbf{x} a_\mathbf{y}} C_{b_\mathbf{x} b_\mathbf{y}} C_{I f_\mathbf{x}} C_{J f_\mathbf{y}}, \end{aligned} \tag{S32}$$

where the truncated double excitation amplitudes $\theta_{f_\mathbf{x} j_\mathbf{x}}^{a_\mathbf{x} b_\mathbf{x}}$ and $\lambda_{a_\mathbf{y} b_\mathbf{y}}^{f_\mathbf{y} j_\mathbf{y}}$ are defined as

$$\theta_{f_\mathbf{x} j_\mathbf{x}}^{a_\mathbf{x} b_\mathbf{x}} = \sum_{i_\mathbf{x}} \theta_{i_\mathbf{x} j_\mathbf{x}}^{a_\mathbf{x} b_\mathbf{x}} C_{i_\mathbf{x} f_\mathbf{x}}, \tag{S33}$$

$$\lambda_{a_\mathbf{y} b_\mathbf{y}}^{f_\mathbf{y} j_\mathbf{y}} = \sum_{i_\mathbf{y}} \lambda_{a_\mathbf{y} b_\mathbf{y}}^{i_\mathbf{y} j_\mathbf{y}} C_{i_\mathbf{y} f_\mathbf{y}}. \tag{S34}$$

And the corresponding transformation matrices are formulated as

$$C_{j_\mathbf{x}j_\mathbf{y}} = \sum_K C_{Kj_\mathbf{x}} C_{Kj_\mathbf{y}}, \tag{S35}$$

$$C_{a_\mathbf{x}a_\mathbf{y}} = \sum_A C_{Aa_\mathbf{x}} C_{Aa_\mathbf{y}}, \tag{S36}$$

$$C_{b_\mathbf{x}b_\mathbf{y}} = \sum_B C_{Bb_\mathbf{x}} C_{Bb_\mathbf{y}}. \tag{S37}$$

Note the double excitation amplitudes should have the symmetry $\theta_{IJ}^{AB} = \theta_{JI}^{BA}$ and $\lambda_{AB}^{IJ} = \lambda_{BA}^{JI}$ for full system, thus, we can rewrite the summation $\sum_{KAB} \theta_{IK}^{AB} \lambda_{AB}^{JK}$ as

$$\sum_{KAB} \theta_{IK}^{AB} \lambda_{AB}^{JK} = \frac{1}{4} \left( \sum_{KAB} \theta_{IK}^{AB} \lambda_{AB}^{JK} + \sum_{KAB} \theta_{KI}^{AB} \lambda_{AB}^{KJ} + \sum_{KAB} \theta_{IK}^{AB} \lambda_{BA}^{JK} + \sum_{KAB} \theta_{KI}^{AB} \lambda_{BA}^{KJ} \right). \tag{S38}$$

Although those 4 summations are equal for the full system, it would be slightly different when using amplitudes constituted from the SIE cluster amplitudes, which are truncated within the fragment, because the symmetry has been broken when truncating the occupied index, shown in Eq. S33 and Eq. S34. To address this problem, the Eq. S32 will be modified based on the equation above, like

$$\begin{aligned}
\sum_{KAB} \theta_{IK}^{AB} \lambda_{AB}^{JK} =& \frac{1}{4} \sum_{\mathbf{xy}} \sum_{f_\mathbf{x} f_\mathbf{y}} \sum_{j_\mathbf{x} j_\mathbf{y}} \sum_{a_\mathbf{x} a_\mathbf{y}} \sum_{b_\mathbf{x} b_\mathbf{y}} \theta_{f_\mathbf{x} j_\mathbf{x}}^{a_\mathbf{x} b_\mathbf{x}} \lambda_{a_\mathbf{y} b_\mathbf{y}}^{f_\mathbf{y} j_\mathbf{y}} C_{j_\mathbf{x} j_\mathbf{y}} C_{a_\mathbf{x} a_\mathbf{y}} C_{b_\mathbf{x} b_\mathbf{y}} C_{I f_\mathbf{x}} C_{J f_\mathbf{y}} \\
&+ \frac{1}{4} \sum_{\mathbf{xy}} \sum_{f_\mathbf{x} f_\mathbf{y}} \sum_{j_\mathbf{x} j_\mathbf{y}} \sum_{a_\mathbf{x} a_\mathbf{y}} \sum_{b_\mathbf{x} b_\mathbf{y}} \theta_{f_\mathbf{x} j_\mathbf{x}}^{a_\mathbf{x} b_\mathbf{x}} \lambda_{a_\mathbf{y} b_\mathbf{y}}^{f_\mathbf{y} j_\mathbf{y}} C_{f_\mathbf{x} f_\mathbf{y}} C_{a_\mathbf{x} a_\mathbf{y}} C_{b_\mathbf{x} b_\mathbf{y}} C_{I j_\mathbf{x}} C_{J j_\mathbf{y}} \\
&+ \frac{1}{4} \sum_{\mathbf{xy}} \sum_{f_\mathbf{x} f_\mathbf{y}} \sum_{j_\mathbf{x} j_\mathbf{y}} \sum_{a_\mathbf{x} a_\mathbf{y}} \sum_{b_\mathbf{x} b_\mathbf{y}} \theta_{f_\mathbf{x} j_\mathbf{x}}^{a_\mathbf{x} b_\mathbf{x}} \lambda_{a_\mathbf{y} b_\mathbf{y}}^{f_\mathbf{y} j_\mathbf{y}} C_{f_\mathbf{x} j_\mathbf{y}} C_{a_\mathbf{x} b_\mathbf{y}} C_{b_\mathbf{x} a_\mathbf{y}} C_{I j_\mathbf{x}} C_{J f_\mathbf{y}} \\
&+ \frac{1}{4} \sum_{\mathbf{xy}} \sum_{f_\mathbf{x} f_\mathbf{y}} \sum_{j_\mathbf{x} j_\mathbf{y}} \sum_{a_\mathbf{x} a_\mathbf{y}} \sum_{b_\mathbf{x} b_\mathbf{y}} \theta_{f_\mathbf{x} j_\mathbf{x}}^{a_\mathbf{x} b_\mathbf{x}} \lambda_{a_\mathbf{y} b_\mathbf{y}}^{f_\mathbf{y} j_\mathbf{y}} C_{j_\mathbf{x} f_\mathbf{y}} C_{a_\mathbf{x} b_\mathbf{y}} C_{b_\mathbf{x} a_\mathbf{y}} C_{I f_\mathbf{x}} C_{J j_\mathbf{y}}.
\end{aligned} \tag{S39}$$

With such rearrangement, the memory usage is reduced from $\mathcal{O}(N^4)$ to $\mathcal{O}(n^3)$, where $n$ represents the size of the SIE clusters, typically no larger than a few hundred orbitals, and normally the number of fragment orbitals is exceedingly small and generally constant. This eliminates the concern of memory pressure. Further simplifications in computation can be achieved by utilizing certain symmetries, such as the need for summation over cluster $\mathbf{x}$ and $\mathbf{y}$, where $\mathbf{x}$ and $\mathbf{y}$ are exchange-symmetrical, thereby allowing calculations for only half of the summation. Additionally, since fragments are selected in real space, spatial symmetries between different fragments may be leveraged to further reduce the computation cost. Moreover, even though the computation of the RDM seems complex, the actual theoretical complexity is at most $\mathcal{O}(n^4)$.

Finally, we give the entire global 1-RDM equation without proving. The occupied-occupied part

denotes

$$\begin{aligned}
\gamma_{IJ} &= 2\delta_{IJ} - P_{IJ}\left(\sum_{A} t_I^A \lambda_A^J + \sum_{KAB} \theta_{IK}^{AB} \lambda_{AB}^{JK}\right) \\
&= 2\delta_{IJ} - P_{IJ}\left(\sum_{A}\left(\sum_{\mathbf{x}}\sum_{f_\mathbf{x} i_\mathbf{x} a_\mathbf{x}} t_{i_\mathbf{x}}^{a_\mathbf{x}} C_{i_\mathbf{x} f_\mathbf{x}} C_{f_\mathbf{x} I} C_{a_\mathbf{x} A}\right)\left(\sum_{\mathbf{y}}\sum_{f_\mathbf{y} i_\mathbf{y} a_\mathbf{y}} \lambda_{a_\mathbf{y}}^{i\mathbf{y}} C_{i_\mathbf{y} f_\mathbf{y}} C_{f_\mathbf{y} I} C_{a_\mathbf{y} A}\right)\right. \\
&\quad + \frac{1}{4}\sum_{\mathbf{xy}}\sum_{f_\mathbf{x} f_\mathbf{y}}\sum_{j_\mathbf{x} j_\mathbf{y}}\sum_{a_\mathbf{x} a_\mathbf{y}}\sum_{b_\mathbf{x} b_\mathbf{y}} \theta_{f_\mathbf{x} j_\mathbf{x}}^{a_\mathbf{x} b_\mathbf{x}} \lambda_{a_\mathbf{y} b_\mathbf{y}}^{f_\mathbf{y} j_\mathbf{y}} C_{j_\mathbf{x} j_\mathbf{y}} C_{a_\mathbf{x} a_\mathbf{y}} C_{b_\mathbf{x} b_\mathbf{y}} C_{I f_\mathbf{x}} C_{J f_\mathbf{y}} \\
&\quad + \frac{1}{4}\sum_{\mathbf{xy}}\sum_{f_\mathbf{x} f_\mathbf{y}}\sum_{j_\mathbf{x} j_\mathbf{y}}\sum_{a_\mathbf{x} a_\mathbf{y}}\sum_{b_\mathbf{x} b_\mathbf{y}} \theta_{f_\mathbf{x} j_\mathbf{x}}^{a_\mathbf{x} b_\mathbf{x}} \lambda_{a_\mathbf{y} b_\mathbf{y}}^{f_\mathbf{y} j_\mathbf{y}} C_{f_\mathbf{x} f_\mathbf{y}} C_{a_\mathbf{x} a_\mathbf{y}} C_{b_\mathbf{x} b_\mathbf{y}} C_{I j_\mathbf{x}} C_{J j_\mathbf{y}} \\
&\quad + \frac{1}{4}\sum_{\mathbf{xy}}\sum_{f_\mathbf{x} f_\mathbf{y}}\sum_{j_\mathbf{x} j_\mathbf{y}}\sum_{a_\mathbf{x} a_\mathbf{y}}\sum_{b_\mathbf{x} b_\mathbf{y}} \theta_{f_\mathbf{x} j_\mathbf{x}}^{a_\mathbf{x} b_\mathbf{x}} \lambda_{a_\mathbf{y} b_\mathbf{y}}^{f_\mathbf{y} j_\mathbf{y}} C_{f_\mathbf{x} j_\mathbf{y}} C_{a_\mathbf{x} b_\mathbf{y}} C_{b_\mathbf{x} a_\mathbf{y}} C_{I j_\mathbf{x}} C_{J f_\mathbf{y}} \\
&\quad \left. + \frac{1}{4}\sum_{\mathbf{xy}}\sum_{f_\mathbf{x} f_\mathbf{y}}\sum_{j_\mathbf{x} j_\mathbf{y}}\sum_{a_\mathbf{x} a_\mathbf{y}}\sum_{b_\mathbf{x} b_\mathbf{y}} \theta_{f_\mathbf{x} j_\mathbf{x}}^{a_\mathbf{x} b_\mathbf{x}} \lambda_{a_\mathbf{y} b_\mathbf{y}}^{f_\mathbf{y} j_\mathbf{y}} C_{j_\mathbf{x} f_\mathbf{y}} C_{a_\mathbf{x} b_\mathbf{y}} C_{b_\mathbf{x} a_\mathbf{y}} C_{I f_\mathbf{x}} C_{J j_\mathbf{y}}\right),
\end{aligned} \tag{S40}$$

and the virtual-virtual part denotes

$$\begin{aligned}
\gamma_{AB} &= P_{AB}\left(\sum_{I} t_I^A \lambda_B^I + \sum_{IJC} \theta_{IJ}^{AC} \lambda_{BC}^{IJ}\right) \\
&= P_{AB}\left(\sum_{I}\left(\sum_{\mathbf{x}}\sum_{f_\mathbf{x} i_\mathbf{x} a_\mathbf{x}} t_{i_\mathbf{x}}^{a_\mathbf{x}} C_{i_\mathbf{x} f_\mathbf{x}} C_{f_\mathbf{x} I} C_{a_\mathbf{x} A}\right)\left(\sum_{\mathbf{y}}\sum_{f_\mathbf{y} i_\mathbf{y} a_\mathbf{y}} \lambda_{a_\mathbf{y}}^{i\mathbf{y}} C_{i_\mathbf{y} f_\mathbf{y}} C_{f_\mathbf{y} I} C_{a_\mathbf{y} A}\right)\right. \\
&\quad + \frac{1}{4}\sum_{\mathbf{xy}}\sum_{f_\mathbf{x} f_\mathbf{y}}\sum_{j_\mathbf{x} j_\mathbf{y}}\sum_{a_\mathbf{x} a_\mathbf{y}}\sum_{b_\mathbf{x} b_\mathbf{y}} \theta_{f_\mathbf{x} j_\mathbf{x}}^{a_\mathbf{x} b_\mathbf{x}} \lambda_{a_\mathbf{y} b_\mathbf{y}}^{f_\mathbf{y} j_\mathbf{y}} C_{f_\mathbf{x} f_\mathbf{y}} C_{j_\mathbf{x} j_\mathbf{y}} C_{b_\mathbf{x} b_\mathbf{y}} C_{A a_\mathbf{x}} C_{B a_\mathbf{y}} \\
&\quad + \frac{1}{4}\sum_{\mathbf{xy}}\sum_{f_\mathbf{x} f_\mathbf{y}}\sum_{j_\mathbf{x} j_\mathbf{y}}\sum_{a_\mathbf{x} a_\mathbf{y}}\sum_{b_\mathbf{x} b_\mathbf{y}} \theta_{f_\mathbf{x} j_\mathbf{x}}^{a_\mathbf{x} b_\mathbf{x}} \lambda_{a_\mathbf{y} b_\mathbf{y}}^{f_\mathbf{y} j_\mathbf{y}} C_{f_\mathbf{x} f_\mathbf{y}} C_{j_\mathbf{x} j_\mathbf{y}} C_{a_\mathbf{x} a_\mathbf{y}} C_{A b_\mathbf{x}} C_{B b_\mathbf{y}} \\
&\quad + \frac{1}{4}\sum_{\mathbf{xy}}\sum_{f_\mathbf{x} f_\mathbf{y}}\sum_{j_\mathbf{x} j_\mathbf{y}}\sum_{a_\mathbf{x} a_\mathbf{y}}\sum_{b_\mathbf{x} b_\mathbf{y}} \theta_{f_\mathbf{x} j_\mathbf{x}}^{a_\mathbf{x} b_\mathbf{x}} \lambda_{a_\mathbf{y} b_\mathbf{y}}^{f_\mathbf{y} j_\mathbf{y}} C_{f_\mathbf{x} j_\mathbf{y}} C_{j_\mathbf{x} f_\mathbf{y}} C_{b_\mathbf{x} a_\mathbf{y}} C_{A a_\mathbf{x}} C_{B b_\mathbf{y}} \\
&\quad \left. + \frac{1}{4}\sum_{\mathbf{xy}}\sum_{f_\mathbf{x} f_\mathbf{y}}\sum_{j_\mathbf{x} j_\mathbf{y}}\sum_{a_\mathbf{x} a_\mathbf{y}}\sum_{b_\mathbf{x} b_\mathbf{y}} \theta_{f_\mathbf{x} j_\mathbf{x}}^{a_\mathbf{x} b_\mathbf{x}} \lambda_{a_\mathbf{y} b_\mathbf{y}}^{f_\mathbf{y} j_\mathbf{y}} C_{f_\mathbf{x} j_\mathbf{y}} C_{j_\mathbf{x} f_\mathbf{y}} C_{a_\mathbf{x} b_\mathbf{y}} C_{A b_\mathbf{x}} C_{B a_\mathbf{y}}\right),
\end{aligned} \tag{S41}$$

and the occupied-virtual part denotes

$$\begin{aligned}
\gamma_{IA} =& t_I^A + \lambda_A^I - \sum_{CK} t_I^C \lambda_C^K t_K^A - \sum_{J} d_{IJ} t_J^A - \sum_{B} d_{AB} t_I^B + \sum_{KC} \theta_{IK}^{AC} \lambda_C^K \\
=& t_I^A + \lambda_A^I - \sum_{CK} t_I^C \lambda_C^K t_K^A - \sum_{J} d_{IJ} t_J^A - \sum_{B} d_{AB} t_I^B \\
&+ \frac{1}{2}\sum_{KC}\sum_{\mathbf{x}}\left(\sum_{k_\mathbf{x} c_\mathbf{x}} \theta_{f_\mathbf{x} k_\mathbf{x}}^{a_\mathbf{x} c_\mathbf{x}} \lambda_C^K C_{K k_\mathbf{x}} C_{C c_\mathbf{x}} C_{I f_\mathbf{x}} C_{A a_\mathbf{x}} + \sum_{k_\mathbf{x} c_\mathbf{x}} \theta_{f_\mathbf{x} k_\mathbf{x}}^{a_\mathbf{x} c_\mathbf{x}} \lambda_C^K C_{K f_\mathbf{x}} C_{C a_\mathbf{x}} C_{I k_\mathbf{x}} C_{A c_\mathbf{x}}\right),
\end{aligned} \tag{S42}$$

where the $t_I^A$ and $\lambda_I^A$ are defined in Eq. S24 and Eq. S25, which should be obtained first before building the global 1-RDM, $d_{IJ} = \sum_{KAB} \theta_{IK}^{AB} \lambda_{AB}^{JK}$ and $d_{AB} = \sum_{IJC} \theta_{IJ}^{AC} \lambda_{BC}^{IJ}$ are the intermediate variables when building the occupied-occupied part and virtual-virtual part in 1-RDM. By following a similar principle shown in Eq. S38, $\sum_{KC} \theta_{IK}^{AC} \lambda_C^K$ could also be split into $\left(\sum_{KC} \theta_{IK}^{AC} \lambda_C^K + \sum_{KC} \theta_{KI}^{CA} \lambda_C^K\right)/2$ then the occupied-virtual part could be rewritten as the last line in the above equation.

### S1.2.2 In-cluster 2-RDM

The key point for building the in-cluster 2-RDM is to use the fragment contracted $\lambda_{a_\mathbf{x}}^{i'_\mathbf{x}}$ and $\lambda_{a_\mathbf{x} b_\mathbf{x}}^{i'_\mathbf{x} j_\mathbf{x}}$ instead of using the standard amplitudes, $\lambda_{a_\mathbf{x}}^{i_\mathbf{x}}$ and $\lambda_{a_\mathbf{x} b_\mathbf{x}}^{i_\mathbf{x} j_\mathbf{x}}$, to build the 2-RDM within the cluster $\mathbf{x}$,

where the $\lambda^{i'_{\mathbf{x}}}_{a_{\mathbf{x}}}$ and $\lambda^{i'_{\mathbf{x}}j_{\mathbf{x}}}_{a_{\mathbf{x}}b_{\mathbf{x}}}$ denote

$$\lambda^{i'_{\mathbf{x}}}_{a_{\mathbf{x}}} = \sum_{i_{\mathbf{x}}} \lambda^{i_{\mathbf{x}}}_{a_{\mathbf{x}}} C^{f_{\mathbf{x}}}_{i_{\mathbf{x}}i'_{\mathbf{x}}}, \tag{S43}$$

$$\lambda^{i'_{\mathbf{x}}j_{\mathbf{x}}}_{a_{\mathbf{x}}b_{\mathbf{x}}} = \sum_{ix} \lambda^{i_{\mathbf{x}}j_{\mathbf{x}}}_{a_{\mathbf{x}}b_{\mathbf{x}}} C^{f_{\mathbf{x}}}_{i_{\mathbf{x}}i'_{\mathbf{x}}}, \tag{S44}$$

where the $C^{f_{\mathbf{x}}}_{i_{\mathbf{x}}i'_{\mathbf{x}}}$ is constructed by contracting the fragment orbitals with the same transformation matrix as

$$C^{f_{\mathbf{x}}}_{i_{\mathbf{x}}i'_{\mathbf{x}}} = \sum_{f_{\mathbf{x}}} C_{i_{\mathbf{x}}f_{\mathbf{x}}} C_{i'_{\mathbf{x}}f_{\mathbf{x}}}, \tag{S45}$$

where the $C_{i_{\mathbf{x}}f_{\mathbf{x}}}$ and $C_{i'_{\mathbf{x}}f_{\mathbf{x}}}$ are the same transformation matrix projecting occupied orbitals of the $\mathbf{x}$ cluster to its fragment orbitals. However, the projected $\lambda^{i'_{\mathbf{x}}j_{\mathbf{x}}}_{a_{\mathbf{x}}b_{\mathbf{x}}}$ would lose the symmetry which means $\lambda^{i'_{\mathbf{x}}j_{\mathbf{x}}}_{a_{\mathbf{x}}b_{\mathbf{x}}} \neq \lambda^{j_{\mathbf{x}}i'_{\mathbf{x}}}_{b_{\mathbf{x}}a_{\mathbf{x}}}$. To keep this symmetry, one approach is

$$\tilde{\lambda}^{i_{\mathbf{x}}j_{\mathbf{x}}}_{a_{\mathbf{x}}b_{\mathbf{x}}} = \frac{1}{2}\left(\lambda^{i'_{\mathbf{x}}j_{\mathbf{x}}}_{a_{\mathbf{x}}b_{\mathbf{x}}} + \lambda^{j_{\mathbf{x}}i'_{\mathbf{x}}}_{b_{\mathbf{x}}a_{\mathbf{x}}}\right), \tag{S46}$$

and use $\tilde{\lambda}^{i_{\mathbf{x}}j_{\mathbf{x}}}_{a_{\mathbf{x}}b_{\mathbf{x}}}$ instead. The subsequent processes for constructing the 2-RDM do not differ from the normal CCSD processing and will not be further elaborated here. It is highly recommended to investigate the CCSD 2-RDM construction for more details see the article [3] and the PySCF code [4, 5].

### S1.2.3 Energy Calculation in PWF-DM

Theoretically, once the 1-RDM and 2-RDM are obtained, all two-body or single-body observables can be computed. Since the focus of the paper is on the energy calculation, here we demonstrate how to use the PWF-DM 1-RDM and 2-RDM for energy computation. The Hamiltonian under Born-Oppenheimer approximation of the entire system can be represented as

$$\hat{H}_e = E_{\text{nuc}} + \sum_{PQ} d_{PQ}\hat{a}^\dagger_P \hat{a}_Q + \frac{1}{2}\sum_{PQRS}(PQ|RS)\hat{a}^\dagger_P \hat{a}^\dagger_Q \hat{a}_S \hat{a}_R \tag{S47}$$

where the $PQRS$ denote the full molecular orbitals, $E_{\text{nuc}}$ is the nuclear repulsion energy and $d_{PQ}$ represents single electron integration coefficients in the molecular basis. Therefore, the total energy for PWF-DM could be obtained as

$$E = E_{\text{nuc}} + \sum_{PQ} d_{PQ}\gamma_{PQ} + \frac{1}{2}\sum_{\mathbf{x}}\sum_{p_{\mathbf{x}}q_{\mathbf{x}}r_{\mathbf{x}}s_{\mathbf{x}}}(p_{\mathbf{x}}q_{\mathbf{x}}|r_{\mathbf{x}}s_{\mathbf{x}})\Gamma_{p_{\mathbf{x}}q_{\mathbf{x}}r_{\mathbf{x}}s_{\mathbf{x}}}, \tag{S48}$$

where the $\gamma_{PQ}$ denotes the global 1-RDM comes from section S1.2.1, and the $\Gamma_{p_{\mathbf{x}}q_{\mathbf{x}}r_{\mathbf{x}}s_{\mathbf{x}}}$ denotes the in-cluster 2-RDM for $\mathbf{x}$ SIE cluster coming from section S1.2.2, the indices $p_{\mathbf{x}}q_{\mathbf{x}}r_{\mathbf{x}}s_{\mathbf{x}}$ denote the molecular orbitals indices within cluster $\mathbf{x}$ and the $(p_{\mathbf{x}}q_{\mathbf{x}}|r_{\mathbf{x}}s_{\mathbf{x}})$ denotes the double electron integration for cluster $\mathbf{x}$, which is projected from full system double electron integration following the equation

$$(p_{\mathbf{x}}q_{\mathbf{x}}|r_{\mathbf{x}}s_{\mathbf{x}}) = \sum_{PQRS}(PQ|RS)C_{Pp_{\mathbf{x}}}C_{Qq_{\mathbf{x}}}C_{Rr_{\mathbf{x}}}C_{Ss_{\mathbf{x}}}. \tag{S49}$$

## S1.3 Perturbative (T) in SIE

### S1.3.1 The In-situ Form

As illustrated in Fig. 1**a**, the SIE+CCSD(T) includes the SIE+MP2/CCSD, perturbative (T), the final treatment of results through the partition wavefunction density matrix (PWF-DM) sections, and the downfolding error correction. Both the SIE embedding part and PWF-DM have been discussed in detail in prior papers [1, 2], with comprehensive formula expressions provided in SI sections S1.1 and S1.2. The downfolding error correction is detailed in SI section S3.7; thus, these topics will not be reiterated here. In this section, we will focus our discussion on the in-cluster form perturbative (T) within SIE.

Our perturbative (T) calculations are performed within each SIE cluster without any amplitude contraction across clusters. Specifically, the closed-shell (T) correlation energy $E_{\mathbf{x}}^{(T)}$ for the $\mathbf{x}$-th SIE cluster can be described by the following formula:

$$E_{\mathbf{x}}^{(T)} = 2 \sum_{(ia \geq jb \geq kc) \in \mathbf{x}} \sum_{i' \in \mathbf{x}} AR\left[Z_{ijk}^{abc}\right] \tilde{W}_{i'jk}^{abc} C_{ii'}^{f}, \tag{S50}$$

where $ijkl$ and $i'$ denote the occupied orbital indices within cluster $\mathbf{x}$, while $abcd$ represents the virtual orbitals. The term $A$ is a conditional constant, expressed as:

$$A = \begin{cases} \frac{1}{6} & \text{if } a = b \text{ and } b = c \\ \frac{1}{2} & \text{if } a = b \text{ or } b = c \\ 1 & \text{else} \end{cases}. \tag{S51}$$

The $Z_{ijk}^{abc}$ denotes

$$Z_{ijk}^{abc} = W_{ijk}^{abc} + V_{ijk}^{abc}, \tag{S52}$$

where $W_{ijk}^{abc}$, $V_{ijk}^{abc}$ and $D_{ijk}^{abc}$ are defined as

$$W_{ijk}^{abc} = \mathcal{P}_{ijk}^{abc}\left[\sum_{l}(ia, bl)t_{kj}^{cl} - \sum_{d}(ia, jd)t_{dk}^{bc}\right] / D_{ijk}^{abc}, \tag{S53}$$

$$V_{ijk}^{abc} = \frac{1}{2}\mathcal{P}_{ijk}^{abc}\left[(ia, jb)t_{k}^{c}\right] / D_{ijk}^{abc}, \tag{S54}$$

$D_{ijk}^{abc}$ and $\tilde{W}_{ijk}^{abc}$ denotes

$$D_{ijk}^{abc} = \epsilon_i + \epsilon_j + \epsilon_k - \epsilon_a - \epsilon_b - \epsilon_c, \tag{S55}$$

$$\tilde{W}_{ijk}^{abc} = W_{ijk}^{abc} D_{ijk}^{abc} \tag{S56}$$

where $t_{ij}^{ab}$ and $t_i^a$ are the single and double excitation amplitudes from CCSD, and $(pq, rs)$ is the electron repulsion integral where $pqrs$ denote occupied or virtual orbital indices here, and $\epsilon_i$ denotes the occupied orbital energy and $\epsilon_a$ denotes the virtual orbital energy. $\mathcal{P}_{ijk}^{abc}$ is the permutation operation,

$$\mathcal{P}_{ijk}^{abc}[X] = X_{ijk}^{abc} + X_{jki}^{bca} + X_{kij}^{cab} + X_{ikj}^{acb} + X_{kji}^{cba} + X_{jik}^{bac}. \tag{S57}$$

And $R$ in eq. S50 is the operation defined as

$$R\left[X\right]_{ijk}^{abc} = 4X_{ijk}^{abc} + X_{klj}^{abc} + X_{jki}^{abc} - 2X_{kji}^{abc} - 2X_{ikj}^{abc} - 2X_{jik}^{abc}. \tag{S58}$$

The $C_{ii'}^{f}$ is the occupied-occupied orbital coefficient truncated by fragment orbitals in $\mathbf{x}$ cluster, which is defined as

$$C_{ii'}^{f} = \sum_{f \in \mathbf{x}} C_{if} C_{i'f}, \tag{S59}$$

where $f$ denotes the fragment orbitals indices (described in SI section S1.1 for detail) in the SIE cluster $\mathbf{x}$. The total correlation energy contributed by perturbative (T) correction could be directly summed over all (T) correlation energy in each SIE cluster,

$$E^{(T)} = \sum_{\mathbf{x}} E_{\mathbf{x}}^{(T)}. \tag{S60}$$

Even though the formula involves a sixth-order tensor like $W_{ijk}^{abc}$ and $V_{ijk}^{abc}$, thanks to the divisibility of the contraction in this form, the actual programming implementation maintains an efficient memory usage, which does not exceed the memory and storage requirements of CCSD.

### S1.3.2 The Ex-situ Form

In the ex-situ form, global 1-RDM and in-cluster 2-RDM will be constructed for perturbative (T). The construction of the global 1-RDM involves interacting information from different SIE clusters, therefore a portion of the perturbation (T) calculations occurs outside of the high-level processing of the SIE clusters, hence being termed the ex-situ form.

For global 1-RDM, the correction from perturbative (T) is given by

$$\Delta\gamma_{IJ} = -\frac{1}{2}\sum_{\mathbf{xy}}\sum_{f_\mathbf{x}}\sum_{f_\mathbf{y}}\sum_{j_\mathbf{x}j_\mathbf{y}}\sum_{k_\mathbf{x}k_\mathbf{y}}\sum_{a_\mathbf{x}a_\mathbf{y}}\sum_{b_\mathbf{x}b_\mathbf{y}}\sum_{c_\mathbf{x}c_\mathbf{y}} (W+V)^{a_\mathbf{x}b_\mathbf{x}c_\mathbf{x}}_{f_\mathbf{x}j_\mathbf{x}k_\mathbf{x}} R\left[W\right]^{a_\mathbf{y}b_\mathbf{y}c_\mathbf{y}}_{f_\mathbf{y}j_\mathbf{y}k_\mathbf{y}} C_{j_\mathbf{x}j_\mathbf{y}} C_{k_\mathbf{x}k_\mathbf{y}} C_{a_\mathbf{x}a_\mathbf{y}} C_{b_\mathbf{x}b_\mathbf{y}} C_{c_\mathbf{x}c_\mathbf{y}} C_{f_\mathbf{x}I} C_{f_\mathbf{y}J}, \tag{S61}$$

$$\Delta\gamma_{AB} = \frac{1}{2}\sum_{\mathbf{xy}}\sum_{a_\mathbf{x}}\sum_{a_\mathbf{y}}\sum_{f_\mathbf{x}f_\mathbf{y}}\sum_{j_\mathbf{x}j_\mathbf{y}}\sum_{k_\mathbf{x}k_\mathbf{y}}\sum_{b_\mathbf{x}b_\mathbf{y}}\sum_{c_\mathbf{x}c_\mathbf{y}} (W+V)^{a_\mathbf{x}b_\mathbf{x}c_\mathbf{x}}_{f_\mathbf{x}j_\mathbf{x}k_\mathbf{x}} R\left[W\right]^{a_\mathbf{y}b_\mathbf{y}c_\mathbf{y}}_{f_\mathbf{y}j_\mathbf{y}k_\mathbf{y}} C_{f_\mathbf{x}f_\mathbf{y}} C_{j_\mathbf{x}j_\mathbf{y}} C_{k_\mathbf{x}k_\mathbf{y}} C_{b_\mathbf{x}b_\mathbf{y}} C_{c_\mathbf{x}c_\mathbf{y}} C_{a_\mathbf{x}A} C_{a_\mathbf{y}B}, \tag{S62}$$

$$\Delta\gamma_{AI} = \frac{1}{2}\sum_{\mathbf{xy}}\sum_{k_\mathbf{x}}\sum_{c_\mathbf{y}}\sum_{f_\mathbf{x}f_\mathbf{y}}\sum_{j_\mathbf{x}j_\mathbf{y}}\sum_{a_\mathbf{x}a_\mathbf{y}}\sum_{b_\mathbf{x}b_\mathbf{y}} t^{a_\mathbf{x}b_\mathbf{x}}_{f_\mathbf{x}j_\mathbf{x}} R\left[W\right]^{a_\mathbf{y}b_\mathbf{y}c_\mathbf{y}}_{f_\mathbf{y}j_\mathbf{y}k_\mathbf{y}} C_{f_\mathbf{x}f_\mathbf{y}} C_{j_\mathbf{x}j_\mathbf{y}} C_{b_\mathbf{x}b_\mathbf{y}} C_{k_\mathbf{x}I} C_{c_\mathbf{y}A}, \tag{S63}$$

where $(W+V)^{a_\mathbf{x}b_\mathbf{x}c_\mathbf{x}}_{f_\mathbf{x}j_\mathbf{x}k_\mathbf{x}}$, $R\left[W\right]^{a_\mathbf{y}b_\mathbf{y}c_\mathbf{y}}_{f_\mathbf{y}j_\mathbf{y}k_\mathbf{y}}$ and $t^{a_\mathbf{x}b_\mathbf{x}}_{f_\mathbf{x}j_\mathbf{x}}$ are the $(W+V)^{a_\mathbf{x}b_\mathbf{x}c_\mathbf{x}}_{i_\mathbf{x}j_\mathbf{x}k_\mathbf{x}}$, $R\left[W\right]^{a_\mathbf{y}b_\mathbf{y}c_\mathbf{y}}_{i_\mathbf{y}j_\mathbf{y}k_\mathbf{y}}$ and $t^{a_\mathbf{x}b_\mathbf{x}}_{i_\mathbf{x}j_\mathbf{x}}$ with their first occupied indices truncated into their SIE cluster fragment, as follows

$$(W+V)^{a_\mathbf{x}b_\mathbf{x}c_\mathbf{x}}_{f_\mathbf{x}j_\mathbf{x}k_\mathbf{x}} = \sum_{i_\mathbf{x}\in\mathbf{x}} C_{i_\mathbf{x}f_\mathbf{x}} (W+V)^{a_\mathbf{x}b_\mathbf{x}c_\mathbf{x}}_{i_\mathbf{x}j_\mathbf{x}k_\mathbf{x}}, \tag{S64}$$

$$R\left[W\right]^{a_\mathbf{y}b_\mathbf{y}c_\mathbf{y}}_{f_\mathbf{y}j_\mathbf{y}k_\mathbf{y}} = \sum_{i_\mathbf{y}\in\mathbf{y}} C_{i_\mathbf{y}f_\mathbf{y}} R\left[W\right]^{a_\mathbf{y}b_\mathbf{y}c_\mathbf{y}}_{i_\mathbf{y}j_\mathbf{y}k_\mathbf{y}}, \tag{S65}$$

$$t^{a_\mathbf{x}b_\mathbf{x}}_{f_\mathbf{x}j_\mathbf{x}} = \sum_{i_\mathbf{x}\in\mathbf{x}} C_{i_\mathbf{x}f_\mathbf{x}} t^{a_\mathbf{x}b_\mathbf{x}}_{i_\mathbf{x}j_\mathbf{x}}, \tag{S66}$$

where $W$, $V$ and $R$ operation have been introduced in eq. S53, eq. S54 and eq. S58, and $t^{a_\mathbf{x}b_\mathbf{x}}_{i_\mathbf{x}j_\mathbf{x}}$ is the double excitation amplitudes from CCSD. $IJKL$ are refereed as the occupied indices in full system molecular orbitals space, $ABCD$ are the virtual indices in molecular orbitals space. the $i_\mathbf{x}j_\mathbf{x}k_\mathbf{x}l_\mathbf{x}$, $a_\mathbf{x}b_\mathbf{x}c_\mathbf{x}d_\mathbf{x}$ and $f_\mathbf{x}$ are occupied, virtual and fragment orbitals in $\mathbf{x}$ SIE cluster, respectively, and similar for the corner mark being $\mathbf{y}$. $C$ represents the coefficient matrix between different orbitals. For example, the $C_{f_\mathbf{x}I}$ means the coefficient between fragment orbitals in cluster $\mathbf{x}$ and full system occupied orbitals. The coefficient matrix between different SIE cluster orbitals is bridged by using full system molecular orbitals, like

$$C_{i_\mathbf{x}i_\mathbf{y}} = \sum_{I} C_{i_\mathbf{x}I} C_{i_\mathbf{y}I}, \tag{S67}$$

$$C_{a_\mathbf{x}a_\mathbf{y}} = \sum_{A} C_{a_\mathbf{x}A} C_{a_\mathbf{y}A}, \tag{S68}$$

$$C_{f_\mathbf{x}f_\mathbf{y}} = \sum_{I} C_{f_\mathbf{x}I} C_{f_\mathbf{y}I}, \tag{S69}$$

For the in-cluster 2-RDM, the correction from perturbative (T) is as follows

$$\Delta\Gamma_{i_\mathbf{x}j_\mathbf{x}a_\mathbf{x}b_\mathbf{x}} = \frac{1}{2}\sum_{l_\mathbf{x}c_\mathbf{x}b_\mathbf{x}}\sum_{k'_\mathbf{x}} R\left[W\right]^{a_\mathbf{x}b_\mathbf{x}c_\mathbf{x}}_{i_\mathbf{x}j_\mathbf{x}k_\mathbf{x}} t^{c_\mathbf{x}}_{k'_\mathbf{x}} C^{f}_{k_\mathbf{x}k'_\mathbf{x}}, \tag{S70}$$

$$\Delta\Gamma_{i_\mathbf{x}a_\mathbf{x}j_\mathbf{x}k_\mathbf{x}} = -\frac{1}{2}\sum_{k_\mathbf{x}c_\mathbf{x}}\sum_{k'_\mathbf{x}} R\left[2W+V\right]^{a_\mathbf{x}b_\mathbf{x}c_\mathbf{x}}_{i_\mathbf{x}j_\mathbf{x}l_\mathbf{x}} t^{b_\mathbf{x}c_\mathbf{x}}_{k'_\mathbf{x}l_\mathbf{x}} C^{f}_{k_\mathbf{x}k'_\mathbf{x}}, \tag{S71}$$

$$\Delta\Gamma_{i_\mathbf{x}a_\mathbf{x}b_\mathbf{x}c_\mathbf{x}} = \frac{1}{2}\sum_{k_\mathbf{x}d_\mathbf{x}}\sum_{k'_\mathbf{x}} R\left[2W+V\right]^{a_\mathbf{x}b_\mathbf{x}d_\mathbf{x}}_{i_\mathbf{x}j_\mathbf{x}k_\mathbf{x}} t^{d_\mathbf{x}c_\mathbf{x}}_{k'_\mathbf{x}j_\mathbf{x}} C^{f}_{k_\mathbf{x}k'_\mathbf{x}}, \tag{S72}$$

where the $C^f_{k_\mathbf{x}k'_\mathbf{x}}$ definition is the same with eq. S60.

It is noteworthy that in constructing the 1-RDM and 2-RDM for CCSD(T), one should also consider generating many equal contraction path at full system level based on some exchange equivalence principles, which has already been discussed in eq. S38. However, although the total number of contraction paths remains the constant, the highest complexity in constructing RDM in perturbative (T) has already reached $\mathcal{O}(n^7)$. Attempting all contraction paths is still prohibitively expensive and unfeasible. Therefore, this section only lists the RDMs provided by the default contraction path.

It should be emphasized that despite the ex-situ form not employing any solutions from the $\Lambda$ equation of CCSD, such as $\lambda^i_a$ or $\lambda^{ij}_{ab}$, the PWF-DM portion of CCSD indeed utilizes them. Consequently, it becomes necessary to incorporate the standard perturbative (T) procedure during the iterative solving of the CCSD's $\Lambda$ equation and to update the solution accordingly. This step could be realized by following the literature [6].

Although it is believed that the ex-situ form, due to the introduction of interactions between clusters in global 1-RDM building, should possess improved accuracy, the complexity on the disk storage would reach $\mathcal{O}(mn^2_{\text{occ}}n^2_{\text{vir}})$ owing to the global 1-RDM building, where $m$ denotes the total number of the clusters and $n_{\text{occ}}$ ($n_{\text{vir}}$) denotes the number of occupied (virtual) orbitals in cluster. This represents an order of magnitude more expensive than the disk complexity of SIE+CCSD(T) using the in-situ form for perturbative (T) and PWF-DM scheme for CCSD, which is considered as $\mathcal{O}(mn_{\text{occ}}n^2_{\text{vir}})$ because only fragment-projected amplitudes need to be saved. Apparently, the ex-situ form will significantly influence the I/O efficiency, potentially making I/O the bottleneck in computations. A further consideration is the steep cost associated with the ex-situ form of the perturbative (T) correction. As indicated in Eq. S61 and Eq. S62, every pair of clusters entails an $\mathcal{O}(n^7)$ tensor contraction, where $n$ denotes cluster size. Consequently, incorporating the full set of cross-cluster terms would demand a total workload that scales as $\mathcal{O}(m^2n^7)$. By contrast, the in-situ formulation performs the (T) correction independently within each cluster, reducing the overall scaling to $\mathcal{O}(mn^7)$, which is one order lower than the ex-situ form. As detailed in the Sec. S1.6, a single CCSD(T) calculation for a 640-orbital cluster already consumes about 60 hours on a single A100, with the perturbative (T) step dominating the runtime. The additional order-of-magnitude cost imposed by the ex-situ scheme is therefore computationally prohibitive, reinforcing our choice of the in-situ approach.

Data presented in the main text already show that the use of the in-situ form in SIE+CCSD(T) already possesses sufficient accuracy. Therefore, in all calculations presented in this paper, we will be employing the in-situ form of SIE+CCSD(T). However, this does not imply that the ex-situ approach should be abandoned. Perhaps with more engineering effort, the I/O consumption can be overshadowed by the computational consumption of the CPU and GPU, thereby maintaining the overall efficiency of the calculations. And maybe not every pair of clusters necessarily warrants an explicit treatment. A lightweight, low-overhead screening procedure should therefore be required to identify which cross-cluster interactions are worth computing besides the in-cluster pairs. For example, one might restrict the cross-cluster evaluation to nearest-neighbor cluster pairs. The effectiveness of such heuristics, however, remains to be systematically assessed. This aspect will be the subject of our continued research and verification in the future.

## S1.4 Complete Basis Set Extrapolation

We use Dunning's correlation-consistent series of Gaussian basis sets throughout this work. Therefore, we follow the two-point extrapolation scheme proposed in Ref. [7]. The extrapolated HF energy is written as

$$E^{\text{HF}}_\infty = E^{\text{HF}}_n - \frac{E^{\text{HF}}_n - E^{\text{HF}}_{n+1}}{1 - e^{-B}}, \tag{S73}$$

where $B$ is a constant value of 1.637 and $n$ is the $\zeta$ cardinality for basis set. The extrapolated correlation energy follow the formula [8]

$$E^{\text{corr}}_\infty = \frac{n^3E^{\text{corr}}_n - m^3E^{\text{corr}}_m}{n^3 - m^3}, \tag{S74}$$

where $n$ and $m$ are the $\zeta$ cardinality for basis set.

## S1.5 The Choice of BNO Threshold

A smaller BNO threshold generally leads to more accurate outcomes at the expense of increased memory usage. When using the canonical CCSD(T) as a solver, the cluster is limited to 800 orbitals in size due to the out-of-memory (OOM) issue. Therefore, in this work, the guiding principle to set the BNO threshold is as small as possible to make the largest clusters not exceed 800 orbitals. $10^{-8}$ is found to be a suitable value. Note in most adsorption cases, with the same BNO threshold, the cluster size constructed from the fragment in the substrate is usually smaller than that in adsorbate. Therefore, in practice, different thresholds are often set for fragments in the substrate and adsorbate to prevent the OOM issue in CCSD(T) calculations.

## S1.6 Linear Scaling for SIE+CCSD(T)

According to the main workflow of SIE+CCSD(T), the complexity of SIE+CCSD(T) is influenced mainly by three aspects. First, the full system MP2 is used to estimate correlations outside the clusters, and thus, it is typical to perform a canonical MP2 calculation on the full system, which has a computational complexity of $\mathcal{O}(N^5)$ where $N$ represents the full system size. However, if SIE+MP2 with a smaller BNO threshold or other local MP2 methods are employed to estimate correlations outside the clusters, the complexity of this component will be reduced. The second aspect is the BNO building, which is the theoretical complexity in the original SIE, marked by $\mathcal{O}(N^3)$ for a cluster [1], the BNO building cost for all $m$ clusters takes $\mathcal{O}(mN^3)$. Clearly, the BNO building cost is not comparable to the full system MP2 cost, and thus can be omitted in the total scaling of SIE+CCSD(T). The final part comes from the time consumption of CCSD(T); performing a CCSD(T) calculation within a SIE cluster of size $n$ is characterized by a complexity of $\mathcal{O}(n^7)$. If there are $m$ clusters in the SIE calculation, this part of the cost is $\mathcal{O}(mn^7)$. Therefore, a general scaling for SIE+CCSD(T) using full system MP2 can be formulated as

$$O = \overset{\text{Full System MP2}}{\mathcal{O}(N^5)} + \overset{\text{Cluster CCSD(T)}}{\mathcal{O}(mn^7)} . \tag{S75}$$

A remarkable feature of SIE is that the cluster size converges as the system size increases with a fixed BNO threshold. Therefore, in calculations where the system size systematically expands, such as in adsorption energy computations where the surface is enlarged to converge the interacting energy, the actual CCSD(T) time consumption becomes a constant number as $n$ converges. At this point, the complexity shifts from $\mathcal{O}(mn^7)$ to a linear scaling $\mathcal{O}(\mathrm{C}m)$, where C represents the constant CCSD(T) time consumption for a size-converged cluster. Thus, the SIE+CCSD(T) scaling becomes

$$O = \overset{\text{Full System MP2}}{\mathcal{O}(N^5)} + \overset{\text{Cluster CCSD(T)}}{\mathcal{O}(\mathrm{C}m)} . \tag{S76}$$

However, in practical SIE+CCSD(T) calculations, there can be a competition between the components of the scaling. To illustrate this issue, consider the SIE+CCSD(T) calculation for $H_2O$@PAH(6) using the ccECP-cc-pVTZ basis set, which involves 6357 orbitals. The full system canonical MP2 is used to correct the bath truncation error. In Table S1 we list the computational time utilized for the full system MP2, BNO building in a cluster, and the CCSD(T) calculation in a cluster.

Table S1: The time consumption estimated on A100 of full system canonical MP2, one cluster BNO building and one cluster CCSD(T) calculation for $H_2O$@PAH(6) with ccECP-cc-pVTZ basis set.

| | Full system MP2 | Cluster BNO building | Cluster CCSD(T) |
|---|---|---|---|
| Theoretical scaling | $\mathcal{O}(N^5)$ | $\mathcal{O}(N^3)$ for a cluster | $\mathcal{O}(n^7)$ for a cluster |
| Size | 6357 | 6357 | 640 |
| Time consumption | 6 hours | 3 minutes | 60 hours |

As observed, although the full system MP2 theoretically presents higher complexity than the other two parts. its inherently non-iterative, straightforward nature of the MP2 enables its efficient implementation on GPU and ensures that its computational demand remains remarkably low, even for the

system with thousands of orbitals. However, even though the cluster size is only about one-tenth of the entire system size, the seventh power complexity of CCSD(T) leads to the time consumed for a single cluster significantly exceeding the time required for the full system MP2 computation, especially considering that it merely includes the time required for only one cluster. The cumulative time cost for CCSD(T) across $m$ clusters ultimately renders the costs for full system MP2 and BNO building negligible. This dominance of CCSD(T) in the SIE+CCSD(T) setup ensures that the framework exhibits a linear complexity of $\mathcal{O}(\mathrm{C}m)$ in medium-sized systems.

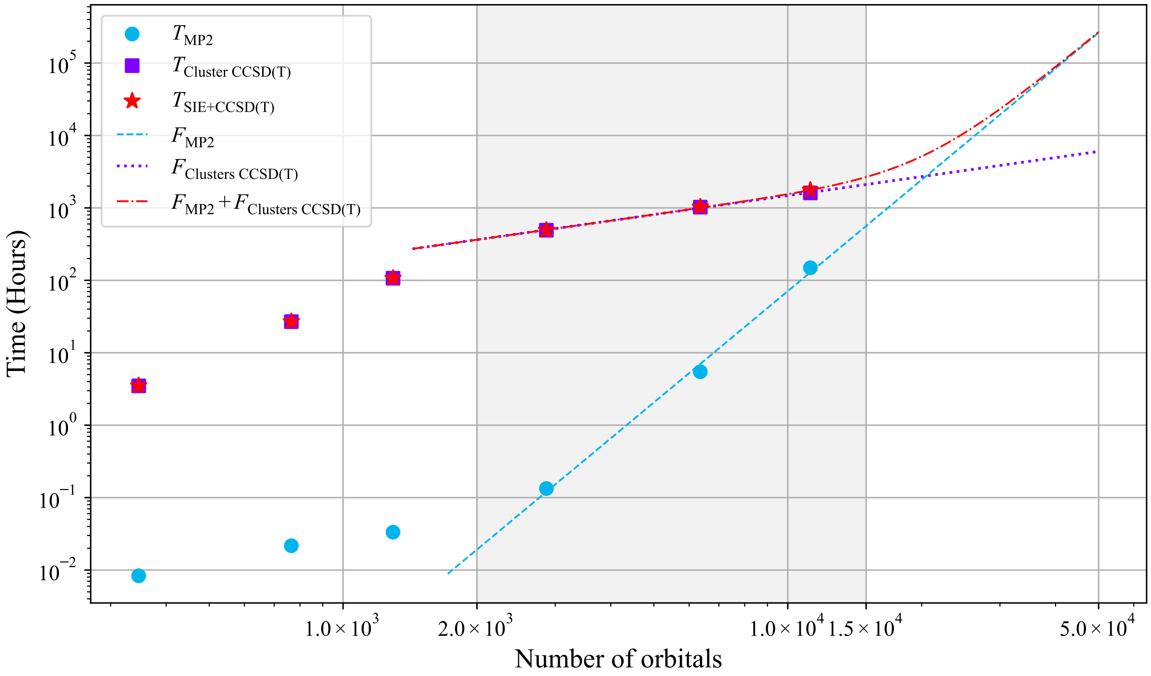


Figure S1: The time consumption for the components in the SIE+CCSD(T) calculations for the $H_2O$@PAH systems. Here, the $T$ denotes the real time cost for the full system MP2 or clusters CCSD(T) in SIE+CCSD(T) or the total SIE+CCSD(T) time consumption. $F$ represents the fitted time cost based on the last 3 real time cost points.

It should be noted that the establishment of linear scaling requires certain conditions. Firstly, for a fixed reasonable BNO threshold, the cluster size must approach convergence so that the time consumed by a single cluster can trend towards a constant. Secondly, when employing full system MP2, it is essential that the MP2 computation does not become the primary consuming component. These conditions imply that the system sizes satisfying linear-scaling SIE+CCSD(T) should fall in an interval. We illustrate this conclusion using the computational cost of $H_2O$@PAH as the system size increases, as illustrated in Figure S1. The MP2 time consumption, $T_{\mathrm{MP2}}$, and the total time consumption of SIE+CCSD(T), $T_{\mathrm{SIE+CCSD(T)}}$, are both depicted in main text Fig. 1**b**. Apart from the time consumed by CCSD(T) in clusters and the full system MP2, other consumption can be disregarded in SIE+CCSD(T). Thus, the difference between $T_{\mathrm{SIE+CCSD(T)}}$ and $T_{\mathrm{MP2}}$ is considered to represent the consumption of clusters CCSD(T), i.e., $T_{\mathrm{Clusters\ CCSD(T)}}$. According to the Figure S1, it is clear that the SIE+CCSD(T) scaling has three stages with the increasing system size. In the first stage, the system is too small, with system size below 2k orbitals. Although the clusters CCSD(T) scaling $\mathcal{O}(mn^7)$ dominates the SIE+CCSD(T), due to the non-convergence of the cluster size, the complexity may slightly exceed linearity. In the second stage, the system size is moderate, as shown by the gray area in the Figure S1 with system sizes ranging from 2k to 15k. In this stage, the consumption of clusters CCSD(T) still remains predominant, but since the cluster size has already converged, the scaling of clusters CCSD(T) reduces to $\mathcal{O}(\mathrm{C}m)$, allowing SIE+CCSD(T) to achieve linear scaling. In the third stage, the system size becomes very large. Since the systems calculated using SIE+CCSD(T) still remain within the linear scaling region, we use the fitted consumption of cluster CCSD(T), $F_{\mathrm{Clusters\ CCSD(T)}}$, combined with the fitted MP2 consumption, $F_{\mathrm{MP2}}$, as the estimation of

total SIE+CCSD(T) cost, $F_{\text{Clusters CCSD(T)}} + F_{\text{MP2}}$. Clearly, when the system size exceeds 15k orbitals, MP2 dominates the scaling of SIE+CCSD(T).

The linear complexity of SIE+CCSD(T) indeed appears in a specific range, and this range is related to factors such as the system type, partition strategy, and BNO threshold. However, it is observed that, for the $H_2O$@Graphene system, this region extends from 2k to 15k orbitals, which is sufficiently broad. This suggests that SIE+CCSD(T) can maintain high efficiency in most problems with a moderate system size.

# S2 GPU-Accelerated High-Performance Package

This section will briefly address a few key components that might become bottlenecks in the SIE computation process, focusing on their general implementation in engineering. For more detailed information, please refer to the code that could already be accessed in github.

## S2.1 SIE framework

In SIE, the computations for each cluster are relatively independent, leading to the use of Message Passing Interface (MPI) to support parallel acceleration computing across multiple GPUs. Furthermore, acceleration of calculations is also supported based on the intrinsic system and partitioning strategy symmetry in real space. That is, if fragment $\mathbf{x}$ and fragment $\mathbf{y}$ are symmetric in space, it is possible to just compute the solution for fragment $\mathbf{x}$, and the solution for fragment $\mathbf{y}$ can be obtained through certain operations transformed from the solution of fragment $\mathbf{x}$.

## S2.2 Electron repulsion integral generation

The Electron Repulsion Integral (ERI) is foundational for performing electronic structure calculations, with the storage consumption scaling as $\mathcal{O}(N^4)$, which is a formidable scaling indeed. For instance, in the largest system we have calculated with 11k orbitals, fully storing the ERI would cost about 104 PB of storage. The density fitting method offers a scheme to reduce the size of ERI, and this trick is used in all the calculations in this paper. After the Cholesky decomposition in density fitting , the storage complexity of ERI (CDERI) is reduced to $\mathcal{O}(N^2 N_{\text{aux}})$, where $N_{\text{aux}}$ represents the size of the auxiliary basis used for density fitting, typically several times the size of the system's total number of orbitals, $N$. Even with density fitting, storing systems with 11k orbitals still requires 20-30 TB of storage space. This extreme storage requirement makes it nearly impossible for ERIs to be stored in running memory or even on disk. However, in SIE, the computations involving ERIs are mostly conducted within subspaces, such as SIE clusters, thus reducing the storage requirement to $\mathcal{O}(mn^2 N_{\text{aux}})$, where $m$ is the number of subspaces, and $n$ is the size of the subspaces.

Even though the CDERI in subspaces should be truncated from the full system's CDERI, it is still unnecessary to store the complete ERI or CDERI. In our software package, the subspace's CDERI is generated on-the-fly. The full system CDERI is produced in slides-by-slides manner and used to generate the subspace's CDERI. Once used, the space taken by the full system CDERI slides would be discarded, reusing the memory to generate the next full system slides. This ensures that the subspace's CDERI can be generated without storing the full system's CDERI, greatly saving on Input/Output cost.

The size of the subspace's CDERI can be further reduced through the use of a truncated auxiliary basis, as the subspace's CDERI still employs the full system's auxiliary basis, which is clearly redundant for the size of the subspace. In theory, singular value decomposition (SVD) can be utilized to directly decompose a subspace $\mathbf{x}$'s CDERI $(L|(p_{\mathbf{x}}q_{\mathbf{x}})_{\text{pair}})$, where $(p_{\mathbf{x}}q_{\mathbf{x}})_{\text{pair}}$ indicates that the indices $p_{\mathbf{x}}q_{\mathbf{x}}$ are paired into a single index. Even though subsystems are small, typically comprising several hundred orbitals, it's often the case that $n^2 \gg N_{\text{aux}}$, leading to a SVD complexity as high as $\mathcal{O}(n^4 N_{\text{aux}})$. Therefore, an alternative path can be sought, as follows

$$(L|L) = \sum_{p_{\mathbf{x}}q_{\mathbf{x}}} (L|p_{\mathbf{x}}q_{\mathbf{x}})(p_{\mathbf{x}}q_{\mathbf{x}}|L), \tag{S77}$$

then diagonalizing the matrix $(L|L)$

$$(L|L) = (L|L_{\mathbf{x}})\boldsymbol{S}^2(L|L_{\mathbf{x}})^{\dagger}, \tag{S78}$$

where $\boldsymbol{S}$ is the singular values for the SVD result of $(L|p_{\mathbf{x}}q_{\mathbf{x}})$ and the $(L|L_{\mathbf{x}})$ is the left singular matrix. A threshold $\kappa$ for $\boldsymbol{S}^2$ could be set to cut off the new dimension $L_{\mathbf{x}}$ like $(L|L_{\mathbf{x}}(\kappa))$. The suggested value for $\kappa$ is $10^{-8}$. Then the new subspace CDERI with truncated auxiliary dimension could be

$$(L_{\mathbf{x}}(\kappa)|p_{\mathbf{x}}q_{\mathbf{x}}) = \sum_{L}(L_{\mathbf{x}}(\kappa)|L)(L|p_{\mathbf{x}}q_{\mathbf{x}}). \tag{S79}$$

Implementing this SVD approach reduces the complexity to $\mathcal{O}(n^2N_{\text{aux}}^2)$. In CCSD and CCSD(T) calculations, the full 4-order ERI is frequently used, leading to many contractions like

$$(p_{\mathbf{x}}q_{\mathbf{x}}|r_{\mathbf{x}}s_{\mathbf{x}}) = \sum_{L_{\mathbf{x}}}(p_{\mathbf{x}}q_{\mathbf{x}}|L_{\mathbf{x}}(\kappa))(L_{\mathbf{x}}(\kappa)|r_{\mathbf{x}}s_{\mathbf{x}}). \tag{S80}$$

Truncating $L_{\mathbf{x}}(\kappa)$ can significantly reduce the computational cost of such calculations, thereby accelerating high-level solver.

In addition, engineering improvement has been done to ensure computational efficiency. The code design incorporates the use of a ping-pong buffer strategy, where memory is divided into two parts. This ensures that computational tasks and I/O tasks alternatively running in the two memory blocks, allowing the time consumed by I/O tasks to be effectively covered by computational time, thereby enhancing overall computational efficiency. Moreover, thanks to this on-the-fly, and slides-by-slides ERI generation mechanism, the processing can be accelerated through parallel processing across multiple GPUs.

### S2.3 High-level solver: MP2

MP2 is utilized extensively within the SIE+CCSD(T) framework, for instance, in the construction of BNOs, in canonical MP2 correction, and sometimes within SIE as a high-level solver. We have made several targeted improvements to our MP2 solver, noting that the relative number of occupied and virtual orbitals, as well as different contraction orders, can significantly impact MP2's memory consumption. Hence, we specifically designed several different schemes for calculating MP2. The ping-pong buffer mechanism has also been introduced to cover the I/O costs between CPU and GPU. Given the simplicity of MP2 principles, it also supports the splitting of matrix contractions, allowing for multi-GPU parallel acceleration of MP2. With those considerable engineering efforts, MP2 can now compute systems with over 11k orbitals, and it has not yet reached its up limit.

### S2.4 High-level solver: CCSD/CCSD(T)

CCSD and CCSD(T) are used as high-level solvers within the SIE framework. Although the CCSD and CCSD(T) are infamous for their prohibitive $\mathcal{O}(N^6)$ and $\mathcal{O}(N^7)$ computational scaling, an even more formidable obstacle in practice is their memory consumption, which directly dictates its feasibility. The challenge is exacerbated on GPUs, whose useable memory is typically limited to only a few tens of GBs. For example, in canonical CCSD, more than ten intermediate tensors scaled as $\mathcal{O}(N^4)$ should be saved in memory. If all of these intermediates were kept resident in GPU memory, the computable system size may be restricted to fewer than 200 orbitals.

To mitigate this bottleneck we adopt a three-pronged strategy. First, the tensor-contraction sequence is rigorously reorganized so that related intermediates are generated, consumed, and discarded within the shortest possible time window. Second, any intermediate that is reused but inexpensive to recompute is generated on-the-fly whenever needed rather than cached. Third, the few indispensable large intermediates are off-loaded to CPU memory, while asynchronous CPU– GPU transfers are scheduled to be overlapped by GPU kernels running, thereby hiding I/O latency. Throughout, strict bookkeeping ensures that both CPU and GPU memory usage remain within predefined limits.

With these optimizations a single A100 GPU can now execute CCSD/CCSD(T) for systems of roughly 700 orbitals, which is sufficient for the vast majority of typical SIE clusters treated in this work.

### S2.5 PWF-DM

The in-cluster 2-RDM from PWF-DM can be computed in-cluster after completing the CCSD calculations without I/O cost. However, the global 1-RDM is not so straightforward, due to the interaction

between clusters. As a result, the amplitudes computed from all clusters must be stored on disk until they are read into CPU and GPU memory for the construction of the global 1-RDM, leading to significant I/O costs between disk, CPU and GPU. Traditional ping-pong buffers often fail to cover these I/O costs due to the considerable size differences between clusters. There might be situation where amplitudes for the next step calculation hasn't been loaded yet while the computations for the previous step are already complete, or the data for the next step is already loaded while the computations for the previous step haven't finished yet, thus wasting either I/O bandwidth or computational resource.

Therefore, we introduced a dedicated memory pool in CPU memory architecture to address this problem. In essence, the pool pre-fetches as many amplitudes from disk as permitted, filling the pool to capacity. While the GPU kernels execute, they draw the required amplitudes directly from this pool; whenever amplitudes are consumed by GPU and the corresponding space is liberated, the next amplitude is streamed in immediately. All transfers are orchestrated asynchronously, and a unified control routine throttles the pre-fetch according to the instantaneous "water level" of the pool. This design hides disk-to-memory latency almost entirely, ensuring that the GPUs remain continuously and efficiently supplied with data.

Besides, the system's inherent real-space symmetry and the exchange symmetry in the eq. S40-S42 between $\mathbf{x}$ and $\mathbf{y}$ are also utilized to reduce the amount of computation.

# S3 Water molecule on graphene

## S3.1 Outline for Achieving OBC-PBC "Handshake"

The adsorption energy of the water@graphene system was calculated using both OBC and PBC models(see Sec. S3.2 for detailed settings of the OBC and PBC structures), achieving an agreement between the OBC and PBC results, i.e., the OBC-PBC "handshake". This is made possible by the SIE+CCSD(T) framework's capability to perform calculations on the large systems and achieve "gold standard" CCSD(T) accuracy. And it also requires the tuning of settings and further corrections tailored to the specific problem.

Firstly, the two hyperparameters of SIE, the BNO threshold and the partition strategy, still require specific settings. We have demonstrated and explained the setting of these two hyperparameters in Sec.S3.3 and Sec S3.7. In addition, the remaining potential errors require further correction.

- **Bath Truncation Error.** This error arises from the truncation of the cluster space. In the Method Sec.4.1, we mentioned that the difference between the full system MP2 and SIE+MP2 can be used to compensate for this part of the error up to the MP2 level. In Sec. S3.7, we will further restrain at the CCSD(T) level in small systems.

- **Basis Set Incompleteness Error.** This error occurs because the Gaussian basis sets used are truncated. The way to compensate is to use two sets of Dunning's correlation-consistent series of Gaussian basis sets and extrapolate the result to the Complete Basis Set (CBS) limit using the extrapolation technique mentioned in Sec. S1.4.

- **Basis Set Superposition Error.** This error occurs when calculating interacting energy and can be eliminated using the counterpoise correction for algorithms employing Gaussian basis sets, which has been discussed in the main text's Method Sec. 4.2.

- **Geometry Relaxation.** This is the error caused by substrate and adsorbate structural relaxation due to the adsorption. We have demonstrated in Sec. S3.9 that DFT can be used to estimate this error.

- **Finite Size Error.** Eliminating this error is key to achieving the OBC-PBC "handshake". This error is caused by the system size being not enough large and it decays as the simulation system size increases. The value or decay form of this finite size error is affected by boundary conditions. By fitting the trend of the results as the OBC/PBC system size increases, we can obtain the result for an infinite large system, which means this result is under the bulk limit condition. Thus, the finite size error is completely eliminated in this result. Please see Sec. S3.8 for detailed information.

After these treatments, the accuracy of the final result should approach the ground truth and be independent of the boundary conditions, thus naturally achieving the OBC-PBC handshake. The estimation of all errors and the derivation of the final adsorption energy are summarized in Sec. S3.10.

## S3.2 Structures

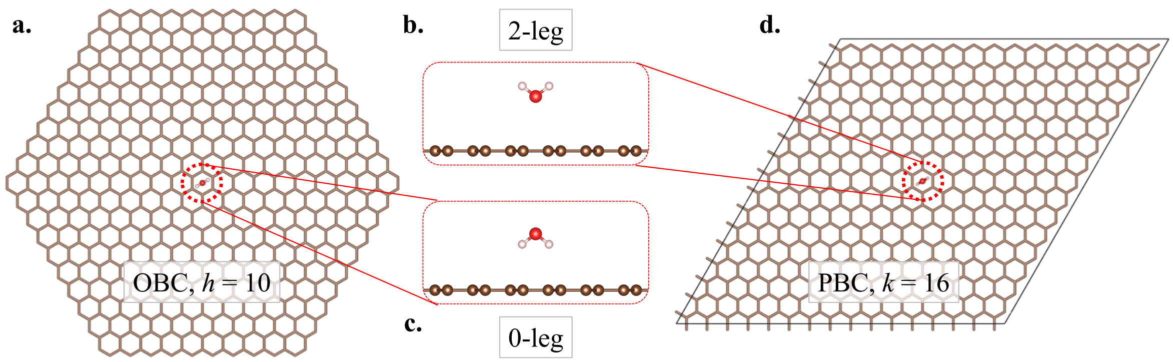


Figure S2: (**a**) OBC structure 0-leg@PAH(10). (**b**), (**c**) is the zoom-in picture for 2-leg configuration in picture (**d**) and 0-leg configuration in picture (**a**), respectively. (**d**) PBC structure 2-leg@Graphene with supercell with $16 \times 16 \times 1$ unit cell.

Various efforts in studying the calculation of adsorption energy for the system water monomer on graphene [9–15], of which two frequently mentioned configurations, 0-leg and 2-leg, are the most likely candidates for the ground-state adsorption configurations. The 0-leg configuration, where two hydrogen atoms from the water monomer are oriented away from the graphene, is shown in Figure S2**b** and the 2-leg configuration, where the hydrogen atoms are oriented towards the graphene, is shown in Figure S2**c**.

In this study, graphene under Open Boundary Conditions (OBC) is modeled using a series of polycyclic aromatic hydrocarbons (PAHs) as substitutes. PAHs can undergo systematic expansion by adding carbon atoms around the periphery. The number of rings denoted by $h$, marks the size of the PAH, PAH($h$), and the molecular formula of PAHs can be expressed as $C_{6h^2}H_{6h}$. Under Periodic Boundary Conditions (PBC), structures were computed at the $\Gamma$ point, and the corresponding overall expansion is achieved by extending the $k \times k \times 1$ supercell at the $\Gamma$ point, containing $2k^2$ carbon atoms. Figure S2 displays the largest systems calculated under the ccECP-cc-pVDZ basis set within this paper, with Figure S2**a** showing PAH(10) calculated under OBC, and Figure S2**b** showing a supercell with $16 \times 16 \times 1$ unit cell under PBC.

Considering that the adsorption energy between water and graphene is very weak, the deformations effect is relatively small. Therefore, in the SIE+CCSD(T) calculations, the configurations employed are those of water and graphene in their independent thermal equilibrium states. Here, we only optimize the distance between the water and graphene and use the equilibrium distance configurations to do the interacting energy calculations. The influence of geometry relaxation will be estimated by DFT. For the detailed information in graphene or PAHs, the bond length is 1.42 Å, and all C-C-C bond angles are 120°. For the peripheral C-H bonds in PAHs, the bond length is 1.089 Å. The O-H bond length within water molecules is 0.957 Å, and the H-O-H bond angle is 104.5°. The geometry relaxation resulting from adsorption is obtained by using DFT and subsequently corrected in the final adsorption energy.

## S3.3 Partition Strategy

The water monomer was considered as an independent fragment, and each carbon atom on the graphene was also treated as a separate fragment. Specially, for OBC, the PAH essentially is a regular hexagon, where all the edge hydrogen atoms on each edge are considered as a single fragment. Therefore, the size of edge hydrogen fragments will grow with increasing PAH size. In Figure S3, we show the partition in $H_2O$@PAH(2) as an example. In this work, the fragments are kept deliberately small.

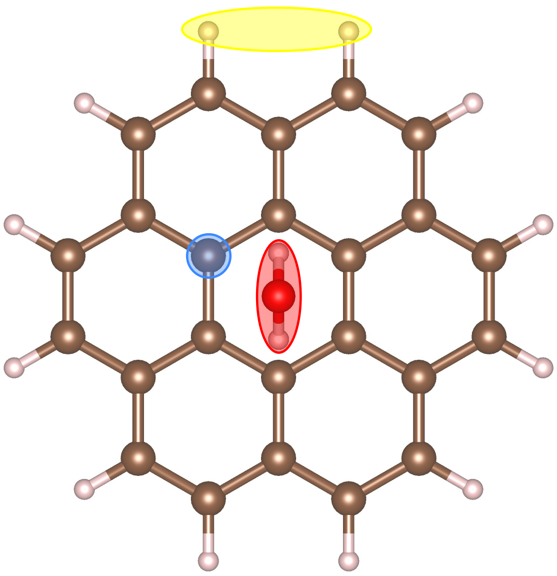

Figure S3: Each differently colored box represents a distinct kind of fragment: the red box denotes the water monomer fragment, the blue box denotes the carbon fragment in graphene, and the yellow box denotes the edge hydrogen atoms fragment.

This choice is made based on an insight that the BNO construction already embeds beyond-mean-field information from MP2, while the fragment and its bath are only constructed at the mean-field level. Expanding a cluster by adding BNOs is therefore markedly more efficient than enlarging the fragment itself. As discussed in the original SIE study [1], see Fig. 3 therein, the BNO expansion accelerates the convergence of the energy relative to fragment expansion. Our strategy is thus to retain the fragments at the smallest reasonable size, where most of the fragments only contain 1 atom. And the clusters are expanded with as many BNOs as practicable, up to the computational limit of our CCSD(T) calculations.

## S3.4 Graphene-Water Distance Optimization

In previous studies, there has been a significant difference in the reported equilibrium distances from the oxygen atom in water monomer to the graphene surface in 2-leg and 0-leg configurations [12, 13, 15]. In table S2, we summarize this equilibrium distances from various works, as well as those obtained after our optimization with SIE+CCSD.

Table S2: The equilibrium distances between the oxygen atom in water monomer and the graphene surface in 2-leg and 0-leg configurations.

| Reference | 0-leg (Å) | 2-leg (Å) |
|---|---|---|
| [12] | 3.06 | 3.20 |
| [15] | 3.075 | 3.155 |
| [13] | 3.10 | 3.37 |
| SIE+CCSD | 3.155 | 3.505 |

During the optimization process, the SIE+CCSD method was utilized, employing the structure of PAH(6), and the partition scheme as described in section S3.3. This approach aimed to obtain relatively accurate interacting energies using the smallest possible basis sets. Therefore a mixed basis set strategy was employed: for the water monomer, the aug-cc-pVDZ basis set was used; for carbon atoms, the cc-pVDZ was selected, and for edge hydrogen atoms, the STO-3G basis set was applied. The BNO threshold for the water monomer fragment was set to $10^{-6.5}$, while for the remaining fragments,

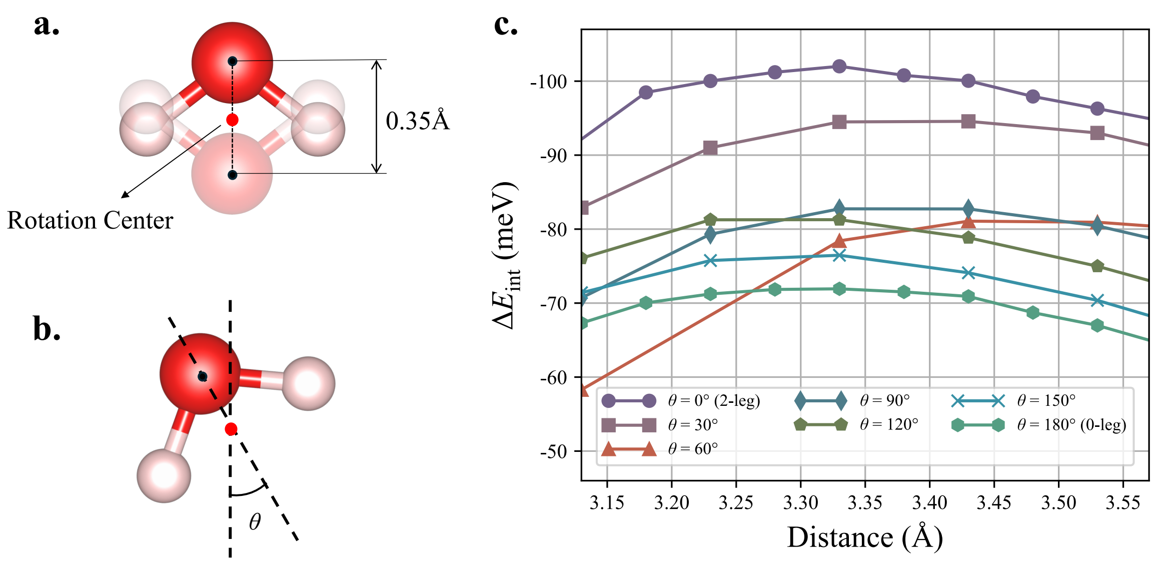


Figure S4: (a) Side views of water monomer in 2-leg and 0-leg configurations. After optimization, the distance between the centers of the oxygen atoms in 2-leg and 0-leg configurations is 0.35 Å. A point located 0.175 Åfrom the oxygen atom in the direction from the negative polarization end towards the positive end is used as the rotation center. Water monomer rotates around the axis which is perpendicular to and through the rotation center. (b) Examples of the water molecule rotating around the rotation center, where $\theta$ refers to the rotational angle relative to the 2-leg configuration. (c) For different $\theta$ configurations, changes in the interacting energy relative to the distance from the rotation center of the monomer to the graphene plane.

it was set to $10^{-8.5}$.

During optimization, the search for the equilibrium distance of the water-graphene in the 2-leg and 0-leg configurations started from 3.155 Å, with a step size of 0.05 Å. Given the sufficiently small step size, the difference in interacting energy between consecutive steps at the equilibrium distance range was very small, within 1 meV. Therefore, we did not perform any further numerical fitting and directly selected the point with the minimum interacting energy as the equilibrium distance for subsequent adsorption energy calculations.

## S3.5 Graphene-Water Distance Optimization under different water orientation

Table S3: $d_{\mathrm{C-G}}$ denotes the equilibrium distance from the rotation center to the graphene surface. And $d_{\mathrm{O-G}}$ denotes the equilibrium distance from O atom in water monomer to the graphene surface.

| $\theta$ | 0° (2-leg) | 30° | 60° | 90° | 120° | 150° | 180° (0-leg) |
|---|---|---|---|---|---|---|---|
| $d_{\mathrm{C-G}}$ (Å) | 3.33 | 3.43 | 3.43 | 3.33 | 3.33 | 3.33 | 3.33 |
| $d_{\mathrm{O-G}}$ (Å) | 3.505 | 3.582 | 3.518 | 3.330 | 3.243 | 3.178 | 3.155 |

For different orientation water on graphene, to ensure consistency in the electronic response induced by the positive and negative polar ends of the rotating water molecule on the graphene surface, we designated the midpoint location of the oxygen atoms in the 0-leg and 2-leg configurations under equilibrium distance as the rotation center, as depicted in Figure S4**a**. The water monomer rotates around an axis passing through the rotation center and perpendicular to the plane of the water monomer. The rotation initiates from the 2-leg configuration and we define the rotation configurations by the angle $\theta$ relative to the 2-leg configuration, as illustrated in Figure S4**b**. Thus, the 2-leg configuration

corresponds to $\theta = 0°$, and the 0-leg configuration to $\theta = 180°$, with additional rotations at theta = 30°, 60°, 90°, 120°, and 150° also considered.

Note these configurations are all optimized to find the equilibrium distance from the rotation center, instead of the O atom in water monomer, to the graphene surface, as shown in the Figure S4**c**. The distance optimization setting is performed as section S3.4 discussed, but with step size of 0.1 Å. All equilibrium distances between the rotation center and the graphene surface are summarized in Table S3. It is important to note that, although the equilibrium distance between the rotation center and the graphene surface is very similar, due to the rotation angle $\theta$, there is still a slight difference in the final equilibrium distance between the oxygen atom in the water monomer and the graphene surface which also be calculated and shown in Table S3.

## S3.6 Interacting Energy Calculation with SIE+CCSD(T)

In the calculations of $H_2O$@Graphene, the BNO threshold for the water monomer fragment is set to $10^{-6.5}$, which is the smallest BNO threshold we could take and results in the adsorbate cluster containing 700 orbitals at most. While for other fragments, it is set to $10^{-8.0}$. The fragmentation used the strategy mentioned in section S3.3, and the distance between the water monomer and graphene plane is fixed with the values mentioned in section S3.4. Basis set used ccECP-cc-pV(D,T)Z (ccECP-(D,T)Z). It is worth noting that, all calculation settings, including the basis set, the distance between water monomer and graphene, the threshold setting and others, are the same for the 0-leg and 2-leg configurations in OBC and PBC. Furthermore, it should be noted that the use of the ccECP basis set is not for the sake of reducing the computational cost. Typically, using the cc-pV$x$Z basis sets for PBC calculations in SIE+CCSD(T) leads to CCSD diverge, whereas the ccECP-cc-pV$x$Z does not encounter this issue. All data are listed in Table. S4-S7, in a unit of meV.

Table S4: Interacting energy (in meV) for 2-leg with OBC.

| Structure | ccECP-DZ | ccECP-TZ | CBS |
|---|---|---|---|
| 2-leg@PAH(2) | -78 | -121 | -138 |
| 2-leg@PAH(4) | -68 | -111 | -128 |
| 2-leg@PAH(6) | -62 | -105 | -122 |
| 2-leg@PAH(8) | -60 | -104 | -121 |
| 2-leg@PAH(10) | -58 | / | / |
| Bulk limit extrapolation | / | / | -114 |

Table S5: Interacting energy (in meV) for 0-leg with OBC.

| Structure | ccECP-DZ | ccECP-TZ | CBS |
|---|---|---|---|
| 0-leg@PAH(2) | 12 | -26 | -40 |
| 0-leg@PAH(4) | -29 | -72 | -90 |
| 0-leg@PAH(6) | -46 | -84 | -100 |
| 0-leg@PAH(8) | -47 | -87 | -104 |
| 0-leg@PAH(10) | -48 | / | / |
| Bulk limit extrapolation | / | / | -107 |

## S3.7 Bath truncation error correction

For any method involving partitioning, there is an inherent error because the subspace size is generally much smaller than the entire system, leading to a poor handling of correlations outside the subspace. Such error stems from the Hamiltonian being truncated to within the subspace. To estimate this error within the SIE framework, a relatively inexpensive correlated method is chosen to measure the

Table S6: Interacting energy (in meV) for 2-leg with PBC.

| Structure | ccECP-DZ | ccECP-TZ | CBS |
|---|---|---|---|
| 2-leg@4 × 4 × 1 | -30 | -65 | -79 |
| 2-leg@8 × 8 × 1 | -51 | -95 | -112 |
| 2-leg@10 × 10 × 1 | -57 | -100 | -116 |
| 2-leg@14 × 14 × 1 | -57 | -99 | -116 |
| 2-leg@16 × 16 × 1 | -58 | / | / |
| Bulk limit extrapolation | / | / | -117 |

Table S7: Interacting energy (in meV) for 0-leg with PBC.

| Structure | ccECP-DZ | ccECP-TZ | CBS |
|---|---|---|---|
| 0-leg@4 × 4 × 1 | -21 | -51 | -64 |
| 0-leg@8 × 8 × 1 | -48 | -83 | -99 |
| 0-leg@10 × 10 × 1 | -50 | -89 | -106 |
| 0-leg@14 × 14 × 1 | -52 | -88 | -104 |
| 0-leg@16 × 16 × 1 | -55 | / | / |
| Bulk limit extrapolation | / | / | -106 |

difference between the result obtained directly using this method for the full system and the result derived using it as a high-level solver for the SIE. MP2 is considered as a suitable choice. Thus, in the main text's method section 4, bath truncation error correction is detailed as

$$\Delta E^{\mathrm{BTEC}}_{\mathrm{MP2}} = E_{\mathrm{MP2}} - E^{\text{PWF-DM}}_{\mathrm{SIE+MP2}}, \tag{S81}$$

where $\Delta E^{\mathrm{BTEC}}_{\mathrm{MP2}}$ denotes the bath truncation error correction at MP2-level, $E_{\mathrm{MP2}}$ denotes the full system MP2 correlation energy, and $E^{\text{PWF-DM}}_{\mathrm{SIE+MP2}}$ denotes the correlation energy calculated by SIE+MP2 with PWF-DM. It is noteworthy that making this correction does not necessarily require a full system MP2 calculation. All methods which as long as can capture more correlation outside the cluster space are acceptable to do this correction. For instance, employing SIE+MP2 results with a smaller BNO threshold is acceptable, which can save some computational resources. In this article, thanks to the efficient engineering implementation of MP2, $E^{\mathrm{BTEC}}_{\mathrm{MP2}}$ is directly obtained from a full system MP2 calculation. In addition, $E^{\text{PWF-DM}}_{\mathrm{SIE+MP2}}$ should utilize the same SIE settings as those used for obtaining $E^{\text{PWF-DM}}_{\mathrm{SIE+CCSD(T)}}$.

The MP2-level bath truncation error correction is considered part of the SIE+CCSD(T) workflow, utilized in all computations within this paper. Unless specifically stated otherwise, it is assumed that this correction has been applied in the results of the SIE+CCSD(T). However, strictly speaking, the bath truncation error of SIE+CCSD(T) should be derived from the difference between full system CCSD(T) and SIE+CCSD(T) results. The correction obtained through MP2 still slightly deviates from the ground truth. Like the results demonstrated in Figure S5, for the $H_2O$@PAH(2) system, although the SIE+CCSD(T) results already include the $\Delta E^{\mathrm{BTEC}}_{\mathrm{MP2}}$, a small gap still remains compared to canonical CCSD(T) outcomes.

Fortunately, for a series of systematically expanded systems, such as the $H_2O$@Graphene system, the CCSD(T)-level bath truncation error correction can be calculated on the smallest system and then applied this correction as a constant to larger systems, thereby enhancing the accuracy of SIE calculation. Specifically, for the OBC $H_2O$@PAH($h$) system, the bath truncation error correction can be estimated as

$$\Delta E^{\mathrm{BTEC}}(\mathrm{OBC}, h) = \Delta E^{\mathrm{BTEC}}_{\mathrm{MP2}}(\mathrm{OBC}, h) + \Delta E^{\mathrm{BTEC}}_{\mathrm{CCSD(T)}}(\mathrm{OBC}, 2), \tag{S82}$$

where $\Delta E^{\mathrm{BTEC}}_{\mathrm{MP2}}(\mathrm{OBC}, h)$ denotes the MP2-level bath truncation error correction for the OBC system $H_2O$@PAH($h$) defined in Eq. S81. And $E^{\mathrm{BTEC}}_{\mathrm{CCSD(T)}}(\mathrm{OBC}, 2)$ denotes improved part of the bath

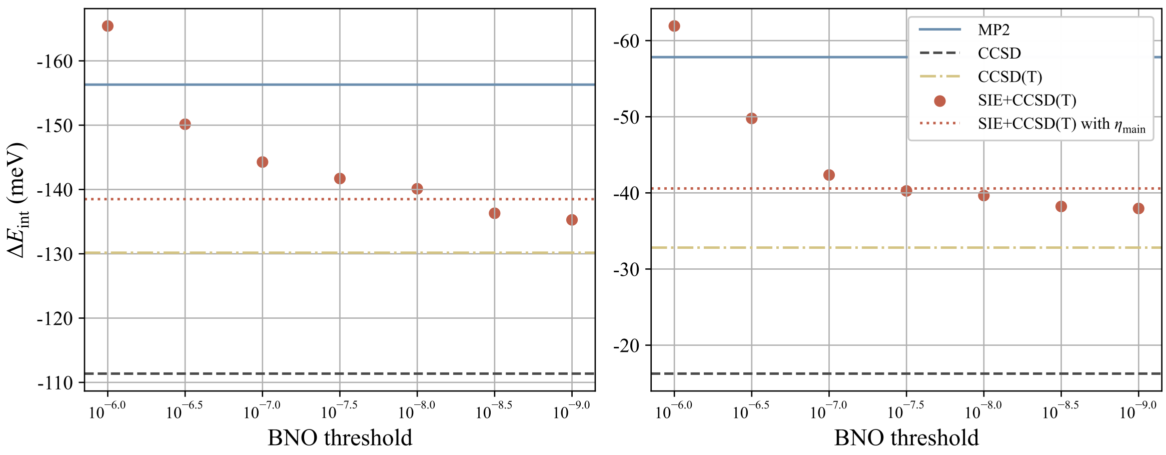


Figure S5: In 2-leg/0-leg@PAH(2), as reducing the BNO threshold, the variation of interacting energy calculated by SIE+CCSD(T) (green solid line) relative to canonical CCSD(T) (red line), canonical CCSD (black line), and canonical MP2 (blue line) are shown. The green dashed line SIE+CCSD(T) with $\eta_{\rm main}$ corresponds to the results calculated using the BNO threshold settings discussed in the main text, which is $10^{-6.5}$ for water monomer fragment and $10^{-8.0}$ for carbon fragments on graphene.

truncation error correction at CCSD(T)-level for $H_2O$@PAH(2), which could be defined as

$$\Delta E^{\rm BTEC}_{\rm CCSD(T)}(\mathrm{OBC},2) = E_{\rm CCSD(T)}(\mathrm{OBC},2,\mathrm{CBS}) - E_{\rm SIE+CCSD(T)}(\mathrm{OBC},2,\mathrm{CBS}), \qquad (S83)$$

where $E_{\rm CCSD(T)}(\mathrm{OBC},2,\mathrm{CBS})$ denotes the canonical CCSD(T) calculated correlation energy under CBS for $H_2O$@PAH(2), and $E_{\rm SIE+CCSD(T)}(\mathrm{OBC},2,\mathrm{CBS})$ denotes the similar correlation energy calculated by SIE+CCSD(T) with MP2-level bath truncation error correction. Since the derivation of interacting energy is based on the direct addition and subtraction of total energies, the bath truncation error correction for interacting energy at the CCSD(T) level can be straightforwardly expressed as

$$\Delta E^{\rm BTEC}_{\rm CCSD(T)}(\mathrm{OBC},2) = \Delta E^{\rm CCSD(T)}_{\rm int}(\mathrm{OBC},2,\mathrm{CBS}) - \Delta E^{\rm SIE+CCSD(T)}_{\rm int}(\mathrm{OBC},2,\mathrm{CBS}), \qquad (S84)$$

where the $\Delta E_{\rm int}$ denotes the interacting energy. The final $\Delta E^{\rm BTEC}_{\rm int,CCSD(T)}(\mathrm{OBC},2)$ values for 2-leg and 0-leg configurations are summarized in Table S8.

Table S8: OBC CCSD(T)-level bath truncation error correction (in meV) for interacting energy estimation on PAH(2).

| Configuration | 2-leg | 0-leg |
|---|---|---|
| $\Delta E^{\rm CCSD(T)}_{\rm int}$ | -130 | -33 |
| $\Delta E^{\rm BTEC}_{\rm CCSD(T)}$ | -138 | -41 |
| $\Delta E^{\rm BTEC}_{\rm CCSD(T)}$ | 8 | 8 |

For PBC, the smallest structure has $4 \times 4 \times 1$ graphene supercell, and under ccECP-TZ basis set the system has nearly 1000 orbitals which is beyond the upper limit we could afford to do the canonical CCSD(T). This issue is addressed by implementing a SIE+CCSD(T) calculation with a smaller threshold,

$$\begin{aligned}\Delta E^{\rm BTEC}_{\rm CCSD(T)}(\mathrm{PBC},4) =& \Delta E^{\rm CCSD(T)}_{\rm int}(\mathrm{PBC},4) - \Delta E^{\rm SIE+CCSD(T)}_{\rm int}(\mathrm{PBC},4,\eta) \\ =& [\Delta E^{\rm CCSD(T)}_{\rm int}(\mathrm{PBC},4) - \Delta E^{\rm SIE+CCSD(T)}_{\rm int}(\mathrm{PBC},4,\eta_s)] \\ &+ [\Delta E^{\rm SIE+CCSD(T)}_{\rm int}(\mathrm{PBC},4,\eta_s) - \Delta E^{\rm SIE+CCSD(T)}_{\rm int}(\mathrm{PBC},4,\eta)]\end{aligned} \qquad (S85)$$

where BNO threshold $\eta_s$ is smaller than $\eta$. The $\eta_s$ is selected to a value such that the SIE+CCSD(T) calculation can be performed under CBS (the largest basis is TZ here) to ensure that the difference

between SIE+CCSD(T) with $\eta_s$ and $\eta$ can be estimated in CBS. While for the difference between canonical CCSD(T) and SIE+CCSD(T) with $\eta_s$, the difference is estimated under DZ basis set which is affordable. Therefore, the formulation could be rewritten as

$$\begin{aligned}\Delta E^{\rm BTEC}_{\rm CCSD(T)}(\text{PBC},4) =&[\Delta E^{\rm CCSD(T)}_{\rm int}(\text{PBC},4,\text{DZ}) - \Delta E^{\rm SIE+CCSD(T)}_{\rm int}(\text{PBC},4,\eta_s,\text{DZ})]\\ &+[\Delta E^{\rm SIE+CCSD(T)}_{\rm int}(\text{PBC},4,\eta_s,\text{CBS}) - \Delta E^{\rm SIE+CCSD(T)}_{\rm int}(\text{PBC},4,\eta,\text{CBS})].\end{aligned} \tag{S86}$$

Here, $\eta_s$ set as $10^{-9.0}$ for all fragments. Then PBC CCSD(T)-level bath truncation error corrections for interacting energy estimated in $H_2O$@Graphene with $4 \times 4 \times 1$ are listed in Table.S9.

Table S9: PBC CCSD(T)-level bath truncation error correction (in meV) for interacting energy estimation on graphene with $4 \times 4 \times 1$ supercell. $\eta_s = 10^{-9.0}$. Unit uses meV.

| Configuration | 2-leg | 0-leg |
|---|---|---|
| $\Delta E^{\rm CCSD(T)}_{\rm int}(\text{DZ})$ | -25 | -16 |
| $\Delta E^{\rm SIE+CCSD(T)}_{\rm int}(\eta_s,\text{DZ})$ | -28 | -19 |
| $\Delta E^{\rm SIE+CCSD(T)}_{\rm int}(\eta_s,\text{CBS})$ | -74 | -58 |
| $\Delta E^{\rm SIE+CCSD(T)}_{\rm int}(\eta,\text{CBS})$ | -79 | -64 |
| $\Delta E^{\rm BTEC}_{\rm CCSD(T)}$ | 8 | 8 |

## S3.8 Bulk Limit Extrapolation

The relationship between the interacting energy and the system size is fitted with the modified nonlinear equation [13],

$$\Delta E_{\rm int} = A + B/d^{\gamma}, \tag{S87}$$

where $A$, $B$, and $\gamma$ are the parameters to be fitted, and $d$ represent the radius of the graphene substrate. The definition of substrate radius for OBC PAH and PBC graphene are shown in Figure. S6. Note, here we use four points for the extrapolation.

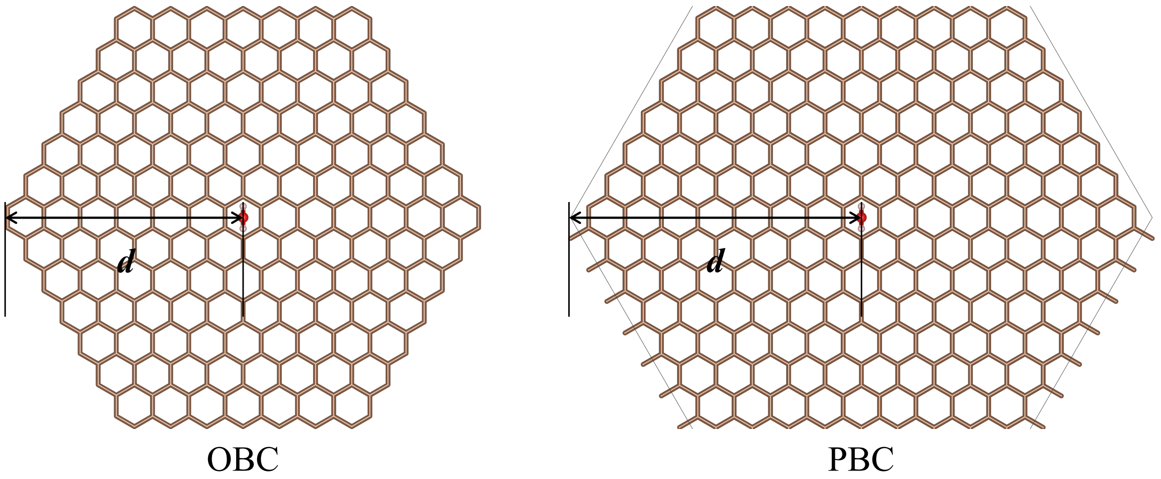


Figure S6: The definitions for radius in OBC and PBC. The edge H atoms are hidden in OBC structure.

With ccECP-TZ basis set, we extended our calculations for OBC up to PAH(8) and for PBC supercell with $14 \times 14 \times 1$ unit cell. With ccECP-DZ basis set, we are able to reach PAH(10) and supercell with $16 \times 16 \times 1$ unit cell, thereby allowing us to verify the reliability of our extrapolations on the ccECP-DZ. All the ccECP-DZ basis set results have been listed in Table S10.The difference

between 4-point and 5-point extrapolated interacting energy on ccECP-DZ basis set would be regarded as the uncertainty estimated by bulk limit extrapolation, which is also listed in the Table. S10.

Figure S7 shows the bulk limit extrapolation of CBS interacting energy of 0-leg and 2-leg under OBC and PBC. The values of the interacting energy at bulk limit could be found in Table S4-S7. Interestingly, we observe that in PBC, for both the 2-leg and 0-leg configurations, the fitted $\gamma$ closely approaches 3, meaning the dipole-dipole interaction between water monomers in different cells is the primary factor influencing the magnitude of finite size error.

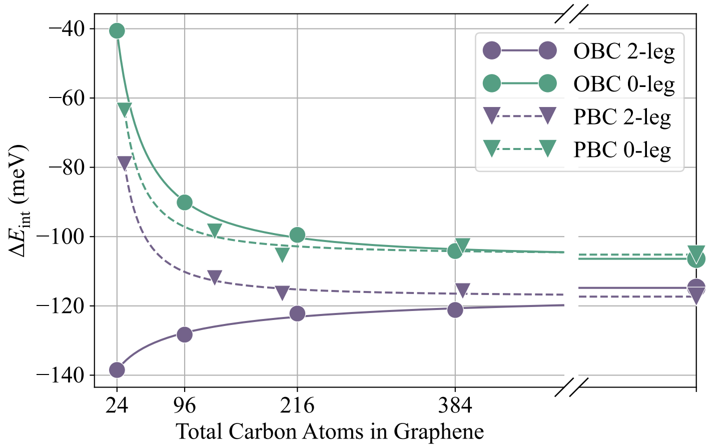


Figure S7: The interacting energy $\Delta E_{\text{int}}$ calculated by SIE+CCSD(T) under ccECP-(D,T)Z extrapolated to CBS.

Table S10: PAH($h$) scales as $C_{6h^2}H_{6h}$ and $k$ stands for the graphene supercell with $k \times k \times 1$ unit cell. The list for $h$ and $k$ is referred as the structures which take part into the bulk limit extrapolation. $\Delta\Delta E_{\text{BL}}$ denotes the uncertainty estimated from bulk limit extrapolation. All energies are in meV.

| | OBC | | | PBC | | |
|---|---|---|---|---|---|---|
| | $h$ in PAH | 2-leg | 0-leg | $k$ in supercell | 2-leg | 0-leg |
| ccECP-DZ | [2,4,6,8] | -49 | -57 | [4,8,10,14] | -62 | -53 |
| | [2,4,6,8,10] | -51 | -53 | [4,8,10,14,16] | -61 | -56 |
| $\Delta\Delta E_{\text{BL}}$ | / | ±3 | ±4 | / | 0 | ±3 |

## S3.9 DFT Study and Geometry Relaxation

The graphene structure and water monomer used in interacting energy calculation are obtained from their respective thermal equilibrium geometries. Although the interaction between graphene and water monomer is relatively weak, the graphene and water monomer may still undergo slight deformations due to adsorption. Therefore, the change in adsorption energy due to this geometry relaxation should be reasonably estimated and included in the overall calculation. Generally, a complete geometry optimization should be performed. However, such optimization is too costly for the current framework of SIE due to the gradient calculation. Therefore, geometry relaxation is estimated at the DFT level.

In an early work [15], multiple DFT functionals are used to calculate the interacting energy of $H_2O$@Graphene. B3LYP+D3, BLYP+D3, PBE0+D3, PBE+D3, revPBE0+D3, revPBE+D3, B97M-V, $\omega$B97M-V are selected by experience from the work [15] to address this task. D3 here is the dispersion correction for functionals [16, 17]. Inspired by this work, we performed all these functionals under cc-pVQZ basis on $H_2O$@PAH(4), which is selected from a series of OBC structures used for SIE+CCSD(T) to calculate interacting energy. The calculations in this section are performed by GPU4PySCF [18–20] and the results are listed in Table S11. As shown in Table S11, the 0-leg configuration of almost all functionals are comparable to SIE+CCSD(T), while for the 2-leg configuration,

only double-hybrid functional B97M-V and $\omega$B97M-V achieved more reasonable results comparable to those of SIE+CCSD(T). Hence, B97M-V and $\omega$B97M-V are employed for the relaxation of the overall structure.

Table S11: The adsorption energies (in meV) for original structures $\Delta E_{\mathrm{org}}$.

| Method | $\Delta E_{\mathrm{org}}^{\text{2-leg}}$ | $\Delta E_{\mathrm{org}}^{\text{0-leg}}$ |
|---|---|---|
| SIE+CCSD(T) | -120 | -82 |
| B3LYP+D3 | -150 | -82 |
| BLYP+D3 | -151 | -79 |
| PBE0+D3 | -144 | -79 |
| PBE+D3 | -146 | -81 |
| revPBE0+D3 | -153 | -73 |
| revPBE+D3 | -160 | -76 |
| B97M-V | -133 | -96 |
| $\omega$B97M-V | -131 | -98 |

We take certain constraints during the structure relaxation process. Firstly, since the effects of adsorption should decrease at the edges, thereby the distances between C-C and C-H on the edges of PAH(4) are fixed during relaxation. Secondly, according to our calculations, all selected functionals greatly underestimated the ground-state distance between graphene and water monomer. To mitigate this issue, the distance between the oxygen atom in the water and the nearest six carbon atoms in PAH(4) was also fixed.

The final adsorption energies for optimized structures are also shown in Table S12. And the adsorption energy differences between the original structure and the optimized structure for functionals are shown in Table S13. The mean value of this difference is used as the geometry relaxation correction, $\Delta E_{\mathrm{geom}}$ to ultimately obtain the adsorption energy, while the root mean square deviation from this average value $\Delta\Delta E_{\mathrm{geom}}$ is considered as the uncertainty of geometry relaxation.

Table S12: The adsorption energies (in meV) for optimized structures $\Delta E_{\mathrm{opt}}$.

| | $\Delta E_{\mathrm{opt}}^{\text{2-leg}}$ | $\Delta E_{\mathrm{opt}}^{\text{0-leg}}$ |
|---|---|---|
| B97M-V | -139 | -103 |
| $\omega$B97M-V | -125 | -94 |

Table S13: The adsorption energy differences (in meV) between different functionals.

| | $\Delta E_{\mathrm{opt}}^{\text{2-leg}} - \Delta E_{\mathrm{org}}^{\text{2-leg}}$ | $\Delta E_{\mathrm{opt}}^{\text{0-leg}} - \Delta E_{\mathrm{org}}^{\text{0-leg}}$ |
|---|---|---|
| B97M-V | -6 | -8 |
| $\omega$B97M-V | 7 | 5 |
| $\Delta E_{\mathrm{geom}}$ | 0 | $-2$ |
| $\Delta\Delta E_{\mathrm{geom}}$ | $\pm 6$ | $\pm 6$ |

## S3.10 $H_2O$@Graphene adsorption energy

As previously mentioned, in the calculations for $H_2O$@Graphene, there are two corrections applied to mitigate the error arising from downfolding Hamiltonian (as discussed in the section S3.7), $\Delta E_{\mathrm{CCSD(T)}}^{\mathrm{BTEC}}$ and the geometry relaxation (as discussed in the section S3.9), $\Delta E_{\mathrm{geom}}$. To correct the bath truncation error, the gap between canonical CCSD(T)and SIE+CCSD(T) is estimated on smallest structure for

Table S14: The final adsorption energy (in meV) with corrections.

| | OBC | | PBC | |
|---|---|---|---|---|
| | 2-leg | 0-leg | 2-leg | 0-leg |
| $\Delta E_{\text{int}}^{\text{SIE+CCSD(T)}}$ | -114 | -107 | -117 | -106 |
| $\Delta E_{\text{CCSD(T)}}^{\text{BTEC}}$ | 8 | 8 | 8 | 8 |
| $\Delta E_{\text{geom}}$ | 0 | $-2$ | 0 | $-2$ |
| $\Delta\Delta E_{\text{BL}}$ | $\pm3$ | $\pm4$ | $\pm0$ | $\pm3$ |
| $\Delta\Delta E_{\text{geom}}$ | $\pm6$ | $\pm6$ | $\pm6$ | $\pm6$ |
| $\Delta E_{\text{ads}}^{\text{SIE+CCSD(T)}}$ | $-106\pm7$ | $-101\pm7$ | $-109\pm6$ | $-100\pm6$ |

OBC PAH(2) and for PBC graphene $4\times4\times1$ supercell. The changes from geometry relaxation are fully studied by DFT using B97M-V and $\omega$B97M-V functionals. Those two corrections are summed up to the SIE+CCSD(T) calculated interacting energy to form the final adsorption energy.

The uncertainty originates from two sources. One is from the bulk limit extrapolation, $\Delta\Delta E_{\text{BL}}$, which comes from using more data to do the bulk limit extrapolation on a smaller basis set, ccECP-DZ (as discussed in the section S3.8). The other source of uncertainty comes from estimating geometry relaxation effect under different functionals, $\Delta\Delta E_{\text{geom}}$ (as discussed in the section S3.9). Those two uncertainties are taken in root-sum-square value to form the total uncertainty for adsorption energy. All components have been summarized in the Table S14, with unit meV.

## S3.11 Interacting energy calculations under different water orientation

This section provides the interacting energies obtained from SIE+CCSD calculations under different water orientations. The SIE+CCSD calculations employed full-system MP2 to correct for bath truncation errors. The BNO threshold settings used in SIE+CCSD are consistent with those used in the optimization of the graphene–water distance, namely $10^{-6.5}$ for the water cluster and $10^{-8.5}$ for the remaining clusters. The cc-pVDZ basis set was used in all calculations. The resulting data are summarized in the following Table S15.

Table S15: Interacting energies (in meV) for different water orientations configurations with OBC. $\theta$ denotes the water rotation angle.

| | PAH(2) | PAH(4) | PAH(6) | PAH(8) |
|---|---|---|---|---|
| 0°(2-leg) | -121 | -106 | -102 | -99 |
| 30° | -109 | -98 | -95 | -93 |
| 60° | -85 | -83 | -81 | -81 |
| 90° | -68 | -80 | -83 | -84 |
| 120° | -45 | -73 | -81 | -83 |
| 150° | -25 | -64 | -77 | -80 |
| 180°(0-leg) | -14 | -59 | -72 | -77 |

## S3.12 Electron density rearrangement

The electron density rearrangement for a adsorption system is defined as

$$\Delta D_e = D_e(\text{AB}) - D_e(\text{A[B]}) - D_e(\text{[A]B}), \tag{S88}$$

where $\Delta D_e$ denotes the electron density rearrangement and $D_e$ denotes the electron density distribution. $D_e(\text{AB})$ denotes the electron density distribution for the full adsorbate-substrate system, $D_e(\text{A[B]})$ denotes the electron density distribution for only adsorbate with substrate being settled as

ghost atom and $D_e$(A[B]) denotes the electron density distribution for only substrate with adsorbate being settled as ghost atom.

The advantage of this definition is that the electron density rearrangement can be considered as only induced by adsorption interactions, excluding other disturbances. In fact, the electron density distribution can be derived using the 1-RDM. Thanks to the specially designed partition wavefunction RDM approach (see more details in S1.2), the global 1-RDM can be obtained via SIE+CCSD, indicating that such electron density rearrangements are characterized with CCSD level accuracy. This undoubtedly provides a more accurate image for understanding adsorption interactions.

## S3.13 Adsorption-Induced Dipole Moment

In the main text, for the configuration $\theta = 60°$, the interacting energy shows negligible variation with increasing PAH size, indicating an fake 'short-range interaction' between water and graphene. This phenomenon is caused by error cancellation. In the main text Fig. 3**b**, the range of electron density rearrangement for configurations with 2-leg, 0-leg, and $\theta = 60°$ shows no significant difference, indicating that the interaction of the $\theta = 60°$ configuration is also long-range. The change in the system's dipole moment caused by adsorption can also provide additional evidence. Here, the change in dipole moment, also named adsorption-induced dipole moment in this paper, $\Delta\mu$, can be defined as

$$\Delta\mu = \mu(\mathrm{AB}) - \mu(\mathrm{A[B]}) - \mu(\mathrm{[A]B}), \tag{S89}$$

where $\mu$(AB) denotes the dipole moment of the combined AB system, while $\mu$(A[B]) denotes the dipole moment in the system comprising A with ghost B, and similarly for $\mu$([A]B). Here, A and B represent the water monomer and the PAH respectively. This definition is very similar to those used for interacting energy (Eq. 4) and electron density rearrangement (Eq. S88). Therefore, $\Delta\mu$ can be considered as entirely introduced due to adsorption.

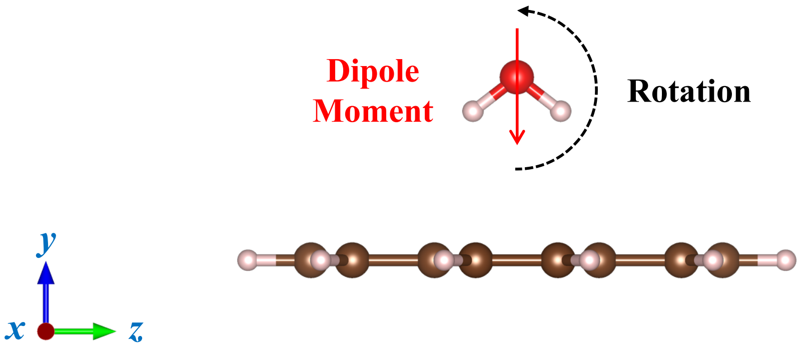


Figure S8: The structure and coordinate axis of 2-leg@PAH(2). The dashed black arrow in the diagram indicates the direction of water rotation. The solid red arrow marks the direction of the dipole moment of the water monomer. Note that the $x$ axis is perpendicular to the plane of the paper and points outside.

The $\Delta\mu$ for $H_2O$@PAH($h$) with $h$ ranging from 2 to 8, were obtained from the global 1-RDM calculated using SIE+CCSD(T) and processed via PySCF. The $\Delta\mu$ components along the $x$, $y$, and $z$ axes are shown in Figure S9. The definition of the $x$, $y$, and $z$ axes, as illustrated, has the water monomer rotating within the $yz$ plane. To more distinctly observe the variation in $\Delta\mu$ with changes in the size of the PAH, we aligned all the curves at zero to the $\Delta\mu$ of $H_2O$@PAH(8), resulting in the definition of $\Delta\Delta\mu$ is

$$\Delta\Delta\mu(h) = \Delta\mu(h) - \Delta\mu(8). \tag{S90}$$

It can be observed that $\Delta\mu_x$ remains almost unchanged with the increasing size of the PAH, which is reasonable since the component of the water monomer dipole moment in the x-axis direction is nearly zero. Additionally, graphene does not spontaneously generate dipole moment along the x-axis, hence $\Delta\mu_x$ is almost zero. However, once the rotation angle of the water deviates from 0° (2-leg) or 180° (0-leg), a component of the dipole moment is generated along the y-axis, and a corresponding

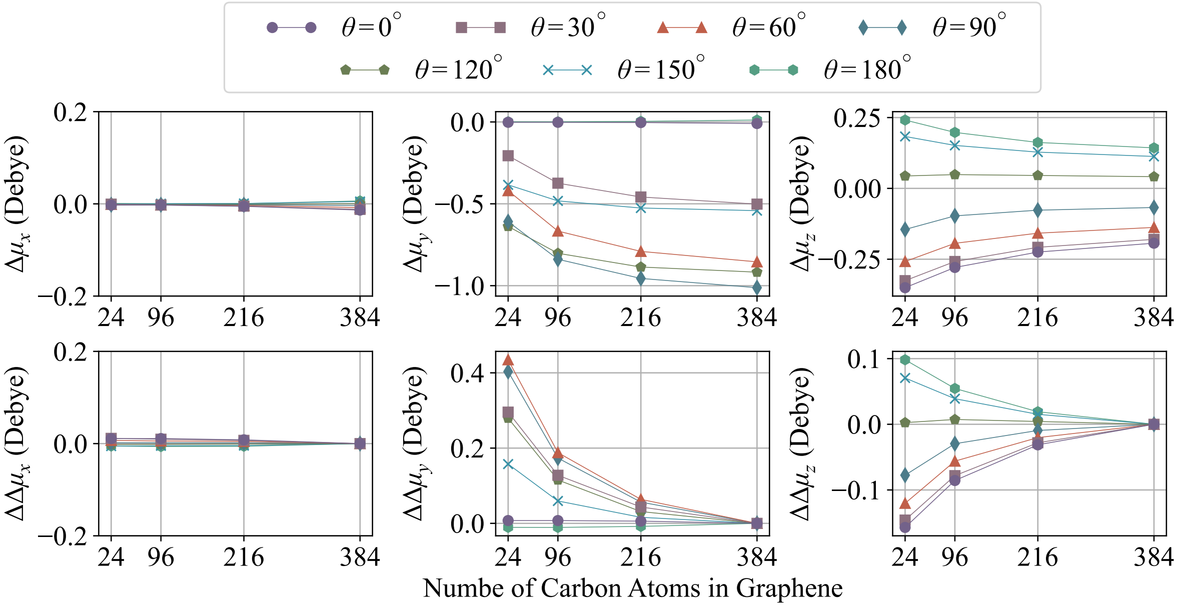


Figure S9: The $\Delta\mu$ and $\Delta\Delta\mu$ components along the $x$, $y$, and $z$ axes changes with the size of PAH increasing from 2 to 8.

opposite dipole moment is induced on the graphene surface along the y-axis to partially counteract the component of the water's dipole moment. From Figure S9**b** (or **f**), it is evident that for the configuration at $\theta = 60°$, $\Delta\mu_y$ (or $\Delta\Delta\mu_y$) changes dramatically with the size of the PAH, highlighting the long-range interaction between water and graphene. A similar conclusion can also be drawn from the changes observed in $\Delta\mu_z$ (or $\Delta\Delta\mu_z$) in Figure S9**c** (or **g**).

## S3.14 Weak Interaction Analysis

As demonstrated in the main text, the water-graphene interaction is insensitive to the orientation of the adsorbed water monomer. Here, we provide additional rationalization using the weak interaction analysis of Independent Gradient Model based on Hirshfeld partitioning (IGMH) [21], a visualization method to illustrate weak interactions. We utilize Multiwfn software [22, 23] to perform IGMH analysis to investigate the interaction regions and types between the water monomer and graphene. We have tested and found that the qualitative features of IGMH analyses are similar to the SIE+CCSD(T) and HF wavefunctions, as compared in Figure S10. Therefore, we provided more detailed comparison of IGMH analyses between different systems with HF wavefunctions computed with cc-pVDZ basis (Figure S11).

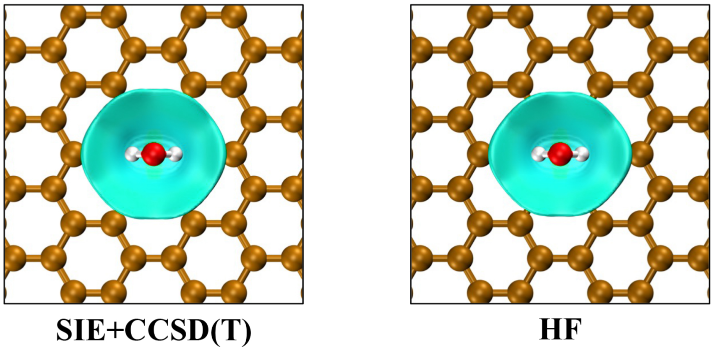


Figure S10: The comparison IGMH analysis between SIE+CCSD(T) and HF results. The figures are the top view of 2-leg@PAH(6).

In the IGMH method, the interaction strength is characterized using a quantity related to electron density, $\delta g^{\mathrm{inter}}$, as detailed in the referenced article [21]. The final IGMH analysis results are shown in the Figure S11. The isosurfaces in the figures represent surfaces of equal interaction strength between adsorbate and substrate, while the colors on these surfaces represent the projection of the interaction strength within the region enclosed by the isosurface. Therefore, if the distribution of interaction strength within the isosurface is uneven, corresponding non-uniform color distributions will appear on the isosurface, with the corresponding color bar provided below the figure. Empirically, green represents van der Waals interactions, blue represents stronger attractive interactions, such as hydrogen bonds, and red represents stronger repulsive interactions.

The most representative configurations—2-leg, 0-leg, and $\theta = 60°$—are analyzed and presented in the first three lines of Figure S11. The isosurfaces are uniformly green, indicating that the interaction strength between water and graphene is consistently even across the board, demonstrating the characteristics of typical van der Waals interactions, and no areas of particularly strong interactions are present. Notably, across different angular configurations, the morphology of the interaction isosurfaces remains similarly bowl-shaped, and there are no significant changes in the intensity or range of interactions.

For comparison, the system of $CH_4$ adsorbed on graphene is also analyzed. $CH_4$, having no polarity due to the complete overlap of its positive and negative polarity centers, exhibits almost identical interacting energies between different adsorption configurations when interacting with the graphene surface [25]. The most representative configurations for $CH_4$@Graphene are 1d and 4d. In the 1d configuration, the carbon atom of $CH_4$ is located above the hole in graphene, with hydrogen atoms directed towards the adjacent carbon atoms on the graphene, whereas in the 4d configuration, $CH_4$'s carbon atom is directly above a carbon atom of graphene, and the hydrogen atoms point toward the holes adjacent in the graphene. In the calculations, graphene is modeled using PAH, and the calculations are done under OBC. For both 1d and 4d configurations, structural optimization are carried out on PAH(4) using $\omega$B97M-V, with all atoms on PAH(4) being fixed. The HF calculations are done by replacing PAH(4) to PAH(6) in the optimized structures. The results, as shown in the last two lines of the Figure S11, clearly show that, the morphology and intensity of interaction region between two $CH_4$@PAH(6) configurations and three $H_2O$@PAH(6) configurations remain highly consistent.

This comparison demonstrates that graphene is indeed capable of canceling out the anisotropy in interactions with water, allowing it to exhibit similar interaction strengths for the configurations across different water rotation configurations. However, this is not commonly observed in studies related to water monomer adsorption. Water is a highly polar molecule and prone to forming hydrogen bond, therefore it usually shows preference towards specific adsorption sites and orientations when interacting with surfaces.

# S4 Carbonaceous molecules on various surfaces

## S4.1 CO@MgO(001)

The CO@MgO(001) adsorption energy calculation primarily follows the approach detailed in a referenced study [26]. The adsorption energy is composed of two parts: interacting energy and geometry relaxation. We directly employ the geometry relaxation from the original study, which is approximately 8 meV, while the interacting energy part is calculated using SIE+CCSD(T). Note that two structures were used to obtain the adsorption energy in the original article. One is 4-Layer model, which contains 4 layers of MgO. The other one is the MgO 2-Layer model, derived from the MgO 4-Layer model. The structures for those two models are shown in Figure S12. The 2-Layer model was employed for p-CCSD(T) in the original paper. The difference between 4-Layer model and 2-Layer model was estimated using MP2 and was regarded as a correction added back to the 2-Layer model p-CCSD (T) results. However, thanks to engineering efforts and the relatively lower complexity of the SIE algorithm, SIE+CCSD(T) does not need to do such things and directly employs the 4-Layer model of MgO(001) for calculations.

During the calculation, CO adsorbed on the substrate is treated as a complete fragment, with the BNO threshold set to $10^{-7.5}$. The remaining fragments in the substrate MgO are obtained by treating each Mg and O atom as a fragment, and using the BNO threshold of $10^{-8.0}$. The BNO thresholds used

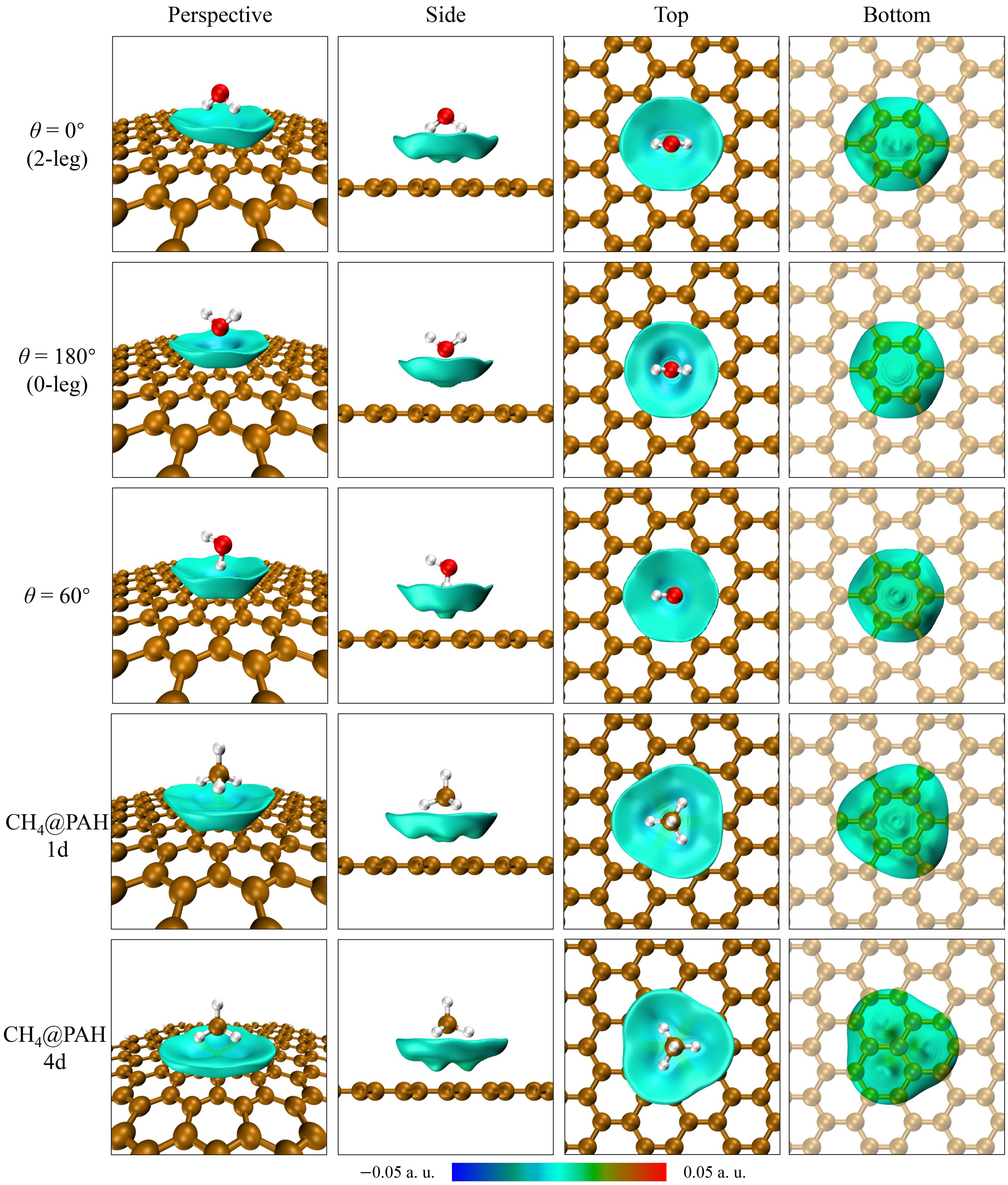


Figure S11: The IGMH weak interaction analysis for 2-leg, 0-leg, $\theta = 60°$ configuration of $H_2O$@PAH(6) (first three lines) and 1d/4d configuration of $CH_4$@PAH(6) (last two lines). The isosurfaces cutoff value for $H_2O$@PAH(6) and $CH_4$@PAH(6) configurations is set as $1 \times 10^{-4}$ a. u. to get a appropriate demonstration for isosurfaces. The color bar here represents the range of interaction strengths projected onto the isosurface, where the same settings are used for all configurations. The structures are rendered by VMD software [24].

in the calculations have essentially reached the upper limit we can afford due to the OOM issue. The basis set employed is the recommended aug-cc-pV$n$Z basis set [27] as the original cluster CCSD(T) used [26]. In this case, we employed a full electron basis set without using any pseudopotentials or frozen core approximations.

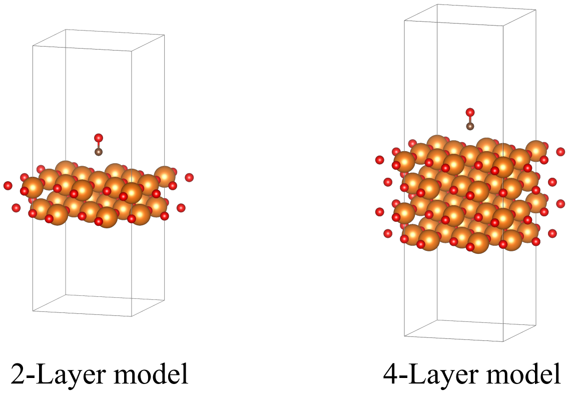


Figure S12: 2-Layer model and 4-Layer model for CO@MgO(001).

Hartree-Fock energy at CBS is obtained through aug-cc-pV(T,Q)Z two-point extrapolation, and correlation energy at CBS is calculated using SIE+CCSD(T) with aug-cc-pV(D,T)Z two-point extrapolation. Finally, the interacting energy and the corresponding adsorption energy are estimated as −213 meV and −205 meV (−4.7 kcal per mol), respectively. Note that the reference adsorption energy calculated by p-CCSD(T) and the experimental data are −193 meV (−4.5 kcal per mol), −198±19 meV (−4.6±0.4 kcal per mol), respectively. In this sense, our method agrees well with these methods within 0.2 kcal per mol.

## S4.2 Organic Molecules@Coronene

Since the optimized structures for the Organic Small Molecules@Coronene composite were not provided in the original article [28], we follow the recent paper [15] and use the double hybrid meta-GGA functional $\omega$B97M-V [29] for the geometry optimization. We first constructed an initial structure roughly based on the structural diagrams presented in the original article's supporting information. Then DFT geometry optimization is conducted using the cc-pVTZ basis set. Initially, optimization was performed using PBE until convergence, and then it was followed by further optimization with $\omega$B97M-V. Note here, we used a model of coronene (PAH(2)) under OBC to represent the substrate graphene as was done in the original paper. SIE+CCSD(T) calculates the interacting energy on the optimized geometries.

However, the experimental data provided is adsorption enthalpy, which cannot directly compare to our adsorption energy. To derive the experimental adsorption energy, the difference offered in the original paper [28] between the optB88-vdW calculated adsorption enthalpy and the interacting energy is used. This difference is subtracted from the experimental adsorption enthalpy to obtain the experimental interacting energy, which then serves as the experimental reference.

In this SIE+CCSD(T) calculation, the cc-pV(D,T)Z basis sets are utilized for basis set extrapolation to CBS. Note that in this case, we treat each atom as an individual fragment for calculations, with a BNO threshold of $10^{-8.0}$. All data have been listed in Table S16, and the energy has been taken as the root-mean-square value and represented in the main text Fig. 4.

In this study, we also investigate whether SIE+CCSD(T) preserves the size consistency. It is well known that MP2 suffers from lacking size consistency due to its omission of higher-order excitations, manifesting that its accuracy cannot be consistently preserved in the systems with different sizes. On the other hand, CCSD and CCSD(T), which employ exponential excitation operators that naturally consider higher-order excitations, are recognized for preserving size consistency. This attribute is a key reason why CCSD and CCSD(T) are regarded as effective for handling large systems. However, since SIE+CCSD(T) is a kind of embedding method, it is worth investigating whether its size consistency can still be maintained.

The group of organic small molecules adsorbed on graphene has gradually increased size, making those adsorption systems suitable for size consistency tests. Specifically, the size of all organic small molecule adsorption systems is labeled by the sum of the atomic numbers of all atoms within the organic small molecules. The interacting energies difference between MP2/SIE+CCSD(T) and experiments

Table S16: Interacting energy (in kcal per mol) comes from SIE+CCSD(T) calculation and experiment.

| Adsorbate | $\Delta E_{\text{int}}^{\text{SIE+CCSD(T)}}$ | $\Delta E_{\text{int}}^{\text{exp}}$ |
|---|---|---|
| Acetone | -8.7 | -8.8 |
| Acetonitrile | -7.0 | -7.4 |
| Dichloromethane | -8.3 | -6.9 |
| Ethanol | -6.5 | -8.7 |
| Ethyl Acetate | -10.7 | -10.8 |
| Toluene | -14.4 | -12.9 |
| RMS | -9.6 | -9.5 |

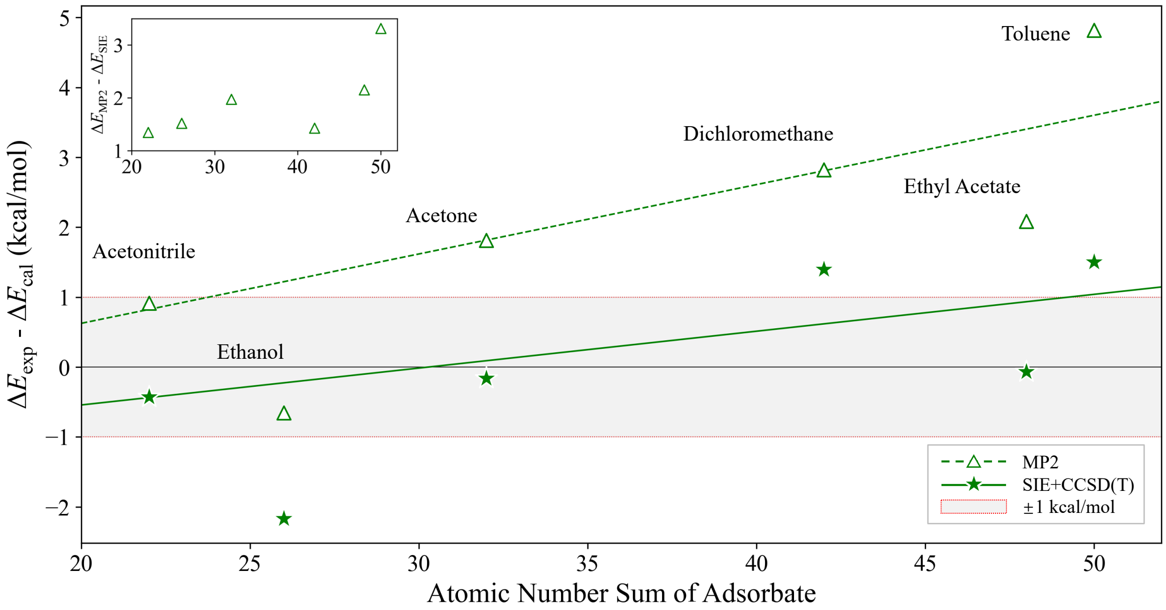


Figure S13: The interacting energies difference between SIE+CCSD(T) or MP2 and experiment for Organic molecules@Coronene. The inset figure shows the interacting energies difference between MP2 and SIE+CCSD(T).

are plotted in Figure S13 related to the size of the system. The relationship between the difference and system size is fitted linearly. Notably, the results for Ethanol are considered as outliers because the SIE+CCSD(T) result deviates further from the experimental value compared to MP2, indicating that there are additional significant errors not considered in the Ethanol@coronene SIE+CCSD(T)/MP2 calculation compared to the real Ethanol@graphene system, such as finite size error. Therefore, the results for Ethanol are not included in the fitting analysis in both MP2 and SIE+CCSD(T).

It can be observed that the slope of the fitting line for SIE+CCSD(T) is significantly lower than that of MP2, suggesting that SIE+CCSD(T) is more size consistent compared to MP2. The same conclusion can be drawn from the difference in interacting energies between MP2 and SIE+CCSD(T), as shown in the inset picture in Figure S13, where the correction by SIE+CCSD(T) to MP2 increases with the system size, indicating that SIE+CCSD(T) strives to maintain size consistency. However, it is worth mentioning that since SIE+CCSD(T) employs MP2 to correct bath truncation errors, the potential for size inconsistency errors may still exist. Considering alternative methods to correct bath truncation errors might avoid this issue, which will be further explored in the future.

## S4.3 $CO/CO_2$@CPO-27-Mg Metal-Organic Framework

The structures are chosen following Ref. [30]. Specifically, CO is adsorbed within a CPO-27-Mg PBC structure that features two large pores, for which the PBE+D2 method is applied for calculation. Then the area close to CO is directly cut out, referred to as the 6**B** cluster, and calculated by MP2 under OBC. Further, a smaller structure, named the 2**B** cluster, is extracted from the 6**B** cluster and subjected to CCSD(T) calculations. The difference between the results from CCSD(T) and MP2 on the 2**B** cluster is employed as a correction, contributing to the total adsorption energy. In our benchmarking case, we choose the most stable adsorption configuration that is the configuration, with the C atom in CO pointing towards the Mg atom in CPO-27-Mg.

Finally, the adsorption energy $\Delta E_{\mathrm{ads}}$ can be expressed as

$$\Delta E_{\mathrm{ads}} = \Delta E^{\mathrm{MP2}}(\mathrm{C}_{6\mathbf{B}}) + \Delta_{\mathrm{LR}}(\mathrm{S}, \mathrm{C}_{6\mathbf{B}}) + \Delta_{\mathrm{CCSD(T)}}(\mathrm{C}_{2\mathbf{B}}) + E_{\mathrm{pair}}, \tag{S91}$$

where the term $\Delta E^{\mathrm{MP2}}(\mathrm{C}_{6\mathbf{B}})$ represents the part of adsorption energy calculated using canonical MP2 for the 6**B** cluster under OBC, $E_{\mathrm{pair}}$ is the pair correction caused by monomer interaction in different adsorption site in PBC structure. $\Delta_{\mathrm{LR}}(\mathrm{S}, \mathrm{C}_{6\mathbf{B}})$ symbolizes the difference between the PBC structure and the OBC 6**B** cluster, utilized to correct a portion of the cluster approach in the 6**B** cluster. $\Delta_{\mathrm{LR}}(\mathrm{S}, \mathrm{C}_{6\mathbf{B}})$ is estimated by PBE+D2. Therefore, $\Delta_{\mathrm{LR}}(\mathrm{S}, \mathrm{C}_{6\mathbf{B}})$ can be expressed as

$$\Delta_{\mathrm{LR}}(\mathrm{S}, \mathrm{C}_{6\mathbf{B}}) = \Delta E^{\mathrm{PBE+D2}}(\mathrm{PBC}) - \Delta E^{\mathrm{PBE+D2}}(\mathrm{OBC}). \tag{S92}$$

$\Delta_{\mathrm{CCSD(T)}}(\mathrm{C}_{2\mathbf{B}})$ is the CCSD(T) correction perform on 2**B** cluster which could be described as

$$\Delta_{\mathrm{CCSD(T)}}(\mathrm{C}_{2\mathbf{B}}) = \Delta E^{\mathrm{CCSD(T)}}(\mathrm{C}_{2\mathbf{B}}) - \Delta E^{\mathrm{MP2}}(\mathrm{C}_{2\mathbf{B}}). \tag{S93}$$

In the case of SIE+CCSD(T), we can handle $\Delta E^{\mathrm{SIE+CCSD(T)}}(\mathrm{C}_{6\mathbf{B}})$ component to replace the $\Delta E^{\mathrm{MP2}}(\mathrm{C}_{6\mathbf{B}}) + \Delta_{\mathrm{CCSD(T)}}(\mathrm{C}_{2\mathbf{B}})$ part of $\Delta E_{\mathrm{ads}}$. However, Our calculated $\Delta E^{\mathrm{MP2}}(\mathrm{C}_{6\mathbf{B}})$, −49.4 kJ/mol, is 4.0 kJ/mol lower than the −45.4 kJ/mol value reported in Ref. [30]. Such small difference may be attributed to only valence shell electrons ($2p$ electrons of Mg have been treated as a valence shell) have been used for MP2 and CCSD(T) calculation in original work [30], but full electrons are used for MP2 and SIE+CCSD(T) calculation in this work. Actually, even for $\Delta E^{\mathrm{SIE+CCSD(T)}}(\mathrm{C}_{6\mathbf{B}})$, the gap is really small compared to the value of $\Delta E^{\mathrm{MP2}}(\mathrm{C}_{6\mathbf{B}}) + \Delta_{\mathrm{CCSD(T)}}(\mathrm{C}_{2\mathbf{B}})$ in original paper. The $\Delta E^{\mathrm{SIE+CCSD(T)}}(\mathrm{C}_{6\mathbf{B}})$ is −46.2 kJ/mol, in comparison the $\Delta E^{\mathrm{MP2}}(\mathrm{C}_{6\mathbf{B}}) + \Delta_{\mathrm{CCSD(T)}}(\mathrm{C}_{2\mathbf{B}})$ is −43.1 kJ/mol, where the absolute difference is 3.1 kJ/mol which is already below than chemical accuracy. To maintain consistency with reference data, we reuse the data provided in the original paper as much as possible. Hence, we chose to calculate $\Delta_{\mathrm{SIE+CCSD(T)}}(\mathrm{C}_{6\mathbf{B}})$ on the 6**B** cluster to replace $\Delta_{\mathrm{CCSD(T)}}(\mathrm{C}_{2\mathbf{B}})$ where $\Delta_{\mathrm{SIE+CCSD(T)}}(\mathrm{C}_{6\mathbf{B}})$ is denoted as

$$\Delta_{\mathrm{SIE+CCSD(T)}}(\mathrm{C}_{6\mathbf{B}}) = \Delta E^{\mathrm{SIE+CCSD(T)}}(\mathrm{C}_{6\mathbf{B}}) - \Delta E^{\mathrm{MP2}}(\mathrm{C}_{6\mathbf{B}}). \tag{S94}$$

The $\Delta_{\mathrm{SIE+CCSD(T)}}(\mathrm{C}_{6\mathbf{B}})$ has the value of 3.2 kJ/mol, combining it with the original $\Delta E^{\mathrm{MP2}}(\mathrm{C}_{6\mathbf{B}})$ (−45.4 kJ/mol), $\Delta_{\mathrm{LR}}(\mathrm{S}, \mathrm{C}_{6\mathbf{B}})$ (−0.1 kJ/mol) and $E_{\mathrm{pair}}$ (−0.3 kJ/mol) in the paper, the adsorption energy with SIE+CCSD(T) correction on 6**B** cluster is −42.4 kJ/mol in agreement with both the original paper, −43.3 kJ/mol and the experiment reference [31] value $-43.8 \pm 1.0$ kJ/mol.

The adsorption energy of $CO_2$@CPO-27-Mg was estimated using the same process. The reference calculated adsorption energy using the isolated model [32] is −50.4 kJ/mol; After a series of corrections, the final adsorption enthalpy was determined to be −41.4 kJ/mol. The difference 9.0 kJ/mol between the calculated adsorption energy and adsorption enthalpy is the zero-point energy and thermal correction and $-RT$ correction. The experimentally measured adsorption enthalpy is $-43 \pm 4$ kJ/mol [33–37]. Thus, the estimated experimental reference adsorption energy is $-52 \pm 4$ kJ/mol after subtracting the zero-point energy, thermal correction and $-RT$ correction.

The $\Delta_{\mathrm{SIE+CCSD(T)}}(\mathrm{C}_{6\mathbf{B}})$ correction obtained using SIE+CCSD(T) on the 6**B** cluster is −1.3 kJ/mol, which is −3.5 kJ/mol less than the previous corrected CCSD(T) value used in [32], Therefore, when using $\Delta_{\mathrm{SIE+CCSD(T)}}(\mathrm{C}_{6\mathbf{B}})$ correction, the adsorption energy value is estimated as −53.9 kJ/mol. In SIE+CCSD(T) calculation, the recommended aug-cc-pV(D,T)Z basis sets are used for basis set extrapolation to CBS. Here, we also treat each atom as an individual fragment for calculations, with a BNO threshold of $10^{-8.0}$.

# S5 Computational details for SIE calculations

Table S17: $H_2O$@Graphene.

| System | $H_2O$@Graphene |
|---|---|
| Boundary condition | OBC and PBC |
| Basis | ccECP-cc-pV(D,T)Z |
| BNO threshold | $10^{-6.5}$ for $H_2O$ $10^{-8.0}$ for graphene |
| Partition | See section S3.3 |

Table S18: CO@MgO(001)

| System | CO@MgO(001) |
|---|---|
| Boundary condition | PBC |
| Basis | aug-cc-pV(T,Q)Z for HF and aug-cc-pV(D,T)Z for SIE |
| BNO threshold | $10^{-7.5}$ for CO $10^{-8.0}$ for MgO |
| Partition | Take CO as a fragment, take every single atom in MgO as a fragment. |

Table S19: Organic molecule @graphene

| System | Organic molecule@graphene |
|---|---|
| Boundary condition | OBC |
| Basis | cc-pV(D,T)Z |
| BNO threshold | $10^{-8.0}$ |
| Partition | Take every single atom as a fragment. |

Table S20: $CO/CO_2$@CPO-27-Mg

| System | $CO/CO_2$@CPO-27-Mg |
|---|---|
| Boundary condition | OBC |
| Basis | aug-cc-pV(D,T)Z |
| BNO threshold | $10^{-8.0}$ |
| Partition | Take every single atom as a fragment. |

## References


[1] Max Nusspickel and George H. Booth. "Systematic Improvability in Quantum Embedding for Real Materials". In: *Physical Review X* 12.1 (2022), p. 011046. ISSN: 2160-3308. DOI: 10.1103/physrevx.12.011046.

[2] Max Nusspickel, Basil Ibrahim, and George H. Booth. "Effective Reconstruction of Expectation Values from Ab Initio Quantum Embedding". In: *Journal of Chemical Theory and Computation* 19.10 (2023), pp. 2769–2791. ISSN: 1549-9618. DOI: 10.1021/acs.jctc.2c01063.

[3] Jürgen Gauss, John F. Stanton, and Rodney J. Bartlett. "Coupled-cluster open-shell analytic gradients: Implementation of the direct product decomposition approach in energy gradient calculations". In: *The Journal of Chemical Physics* 95.4 (1991), pp. 2623–2638. ISSN: 0021-9606. DOI: 10.1063/1.460915.

[4] Qiming Sun et al. "Recent developments in the PySCF program package". In: *The Journal of Chemical Physics* 153.2 (2020). ISSN: 0021-9606. DOI: 10.1063/5.0006074.

[5] Qiming Sun et al. "PySCF: the Python-based simulations of chemistry framework". In: *WIREs Computational Molecular Science* 8.1 (Sept. 2018), e1340. ISSN: 1759-0884. DOI: 10.1002/wcms.1340.

[6] Uğur Bozkaya and C. David Sherrill. "Analytic energy gradients for the coupled-cluster singles and doubles with perturbative triples method with the density-fitting approximation". In: *The Journal of Chemical Physics* 147.4 (July 2017). ISSN: 1089-7690. DOI: 10.1063/1.4994918.

[7] Asger Halkier et al. "Basis-set convergence of the energy in molecular Hartree–Fock calculations". In: *Chemical Physics Letters* 302.5-6 (1999), pp. 437–446. ISSN: 0009-2614. DOI: 10.1016/s0009-2614(99)00179-7.

[8] Asger Halkier et al. "Basis-set convergence in correlated calculations on Ne, $N_2$, and $H_2O$". In: *Chemical Physics Letters* 286.3-4 (1998), pp. 243–252. ISSN: 0009-2614. DOI: 10.1016/s0009-2614(98)00111-0.

[9] Jie Ma et al. "Adsorption and diffusion of water on graphene from first principles". In: *Physical Review B* 84.3 (2011), p. 033402. ISSN: 1098-0121. DOI: 10.1103/physrevb.84.033402.

[10] Miroslav Rubeš et al. "Structure and Stability of the Water-Graphite Complexes". In: *The Journal of Physical Chemistry C* 113.19 (2009), pp. 8412–8419. ISSN: 1932-7447. DOI: 10.1021/jp901410m.

[11] Glen R. Jenness, Ozan Karalti, and Kenneth D. Jordan. "Benchmark calculations of water–acene interaction energies: Extrapolation to the water–graphene limit and assessment of dispersion–corrected DFT methods". In: *Physical Chemistry Chemical Physics* 12.24 (2010), pp. 6375–6381. ISSN: 1463-9076. DOI: 10.1039/c000988a.

[12] Elena Voloshina et al. "On the physisorption of water on graphene: a CCSD(T) study". In: *Physical Chemistry Chemical Physics* 13.25 (2011), p. 12041. ISSN: 1463-9084. DOI: 10.1039/c1cp20609e.

[13] Jan Gerit Brandenburg et al. "Physisorption of Water on Graphene: Subchemical Accuracy from Many-Body Electronic Structure Methods". In: *The Journal of Physical Chemistry Letters* 10.3 (2019), pp. 358–368. ISSN: 1948-7185. DOI: 10.1021/acs.jpclett.8b03679.

[14] Bryan T. G. Lau, Gerald Knizia, and Timothy C. Berkelbach. "Regional Embedding Enables High-Level Quantum Chemistry for Surface Science". In: *The Journal of Physical Chemistry Letters* 12.3 (2021), pp. 1104–1109. ISSN: 1948-7185. DOI: 10.1021/acs.jpclett.0c03274.

[15] Adeayo O. Ajala et al. "Assessment of Density Functional Theory in Predicting Interaction Energies between Water and Polycyclic Aromatic Hydrocarbons: from Water on Benzene to Water on Graphene". In: *Journal of Chemical Theory and Computation* 15.4 (2019), pp. 2359–2374. ISSN: 1549-9618. DOI: 10.1021/acs.jctc.9b00110.

[16] Sándor Kristyán and Péter Pulay. "Can (semi)local density functional theory account for the London dispersion forces?" In: *Chemical Physics Letters* 229.3 (1994), pp. 175–180. ISSN: 0009-2614. DOI: 10.1016/0009-2614(94)01027-7.

[17] Pavel Hobza, Jiří šponer, and Tomáš Reschel. “Density functional theory and molecular clusters”. In: *Journal of Computational Chemistry* 16.11 (1995), pp. 1315–1325. ISSN: 0192-8651. DOI: 10.1002/jcc.540161102.

[18] Xiaojie Wu et al. *Enhancing GPU-acceleration in the Python-based Simulations of Chemistry Framework*. 2024. DOI: 10.48550/ARXIV.2404.09452.

[19] Susi Lehtola et al. “Recent developments in libxc — A comprehensive library of functionals for density functional theory”. In: *SoftwareX* 7 (2018), pp. 1–5. ISSN: 2352-7110. DOI: 10.1016/j.softx.2017.11.002.

[20] Ulf Ekström et al. “Arbitrary-Order Density Functional Response Theory from Automatic Differentiation”. In: *Journal of Chemical Theory and Computation* 6.7 (2010), pp. 1971–1980. ISSN: 1549-9618. DOI: 10.1021/ct100117s.

[21] Tian Lu and Qinxue Chen. “Independent gradient model based on Hirshfeld partition: A new method for visual study of interactions in chemical systems”. In: *Journal of Computational Chemistry* 43.8 (Feb. 2022), pp. 539–555. ISSN: 1096-987X. DOI: 10.1002/jcc.26812.

[22] Tian Lu and Feiwu Chen. “Multiwfn: A multifunctional wavefunction analyzer”. In: *Journal of Computational Chemistry* 33.5 (Dec. 2011), pp. 580–592. ISSN: 1096-987X. DOI: 10.1002/jcc.22885.

[23] Tian Lu. “A comprehensive electron wavefunction analysis toolbox for chemists, Multiwfn”. In: *The Journal of Chemical Physics* 161.8 (2024). ISSN: 0021-9606. DOI: 10.1063/5.0216272.

[24] William Humphrey, Andrew Dalke, and Klaus Schulten. “VMD – Visual Molecular Dynamics”. In: *Journal of Molecular Graphics* 14.1 (1996), pp. 33–38. ISSN: 0263-7855. DOI: 10.1016/0263-7855(96)00018-5.

[25] C. Thierfelder et al. “Methane adsorption on graphene from first principles including dispersion interaction”. In: *Surface Science* 605.7-8 (2011), pp. 746–749. ISSN: 0039-6028. DOI: 10.1016/j.susc.2011.01.012.

[26] Benjamin X. Shi et al. “Many-Body Methods for Surface Chemistry Come of Age: Achieving Consensus with Experiments”. In: *Journal of the American Chemical Society* 145.46 (Nov. 2023), pp. 25372–25381. ISSN: 1520-5126. DOI: 10.1021/jacs.3c09616.

[27] Rick A. Kendall, Thom H. Dunning, and Robert J. Harrison. “Electron affinities of the first-row atoms revisited. Systematic basis sets and wave functions”. In: *The Journal of Chemical Physics* 96.9 (1992), pp. 6796–6806. ISSN: 0021-9606. DOI: 10.1063/1.462569.

[28] Petr Lazar et al. “Adsorption of Small Organic Molecules on Graphene”. In: *Journal of the American Chemical Society* 135.16 (2013), pp. 6372–6377. ISSN: 0002-7863. DOI: 10.1021/ja403162r.

[29] Narbe Mardirossian and Martin Head-Gordon. “$\omega$B97M-V: A combinatorially optimized, range-separated hybrid, meta-GGA density functional with VV10 nonlocal correlation”. In: *The Journal of Chemical Physics* 144.21 (2016). ISSN: 0021-9606. DOI: 10.1063/1.4952647.

[30] Arpan Kundu et al. “Ab Initio Prediction of Adsorption Isotherms for Small Molecules in Metal–Organic Frameworks”. In: *Journal of the American Chemical Society* 138.42 (2016), pp. 14047–14056. ISSN: 0002-7863. DOI: 10.1021/jacs.6b08646.

[31] Joachim Sauer. “Ab Initio Calculations for Molecule–Surface Interactions with Chemical Accuracy”. In: *Accounts of Chemical Research* 52.12 (2019), pp. 3502–3510. ISSN: 0001-4842. DOI: 10.1021/acs.accounts.9b00506.

[32] Kaido Sillar and Ivar Koppel. “Ab Initio Calculation of Thermodynamic Functions for $CO_2$ Adsorption in Metal–Organic Frameworks: Entropic Effects of Lateral Interactions”. In: *The Journal of Physical Chemistry C* 127.24 (2023), pp. 11712–11719. ISSN: 1932-7447. DOI: 10.1021/acs.jpcc.3c02234.

[33] Wendy L. Queen et al. “Comprehensive study of carbon dioxide adsorption in the metal–organic frameworks $M_2$(dobdc) (M = Mg, Mn, Fe, Co, Ni, Cu, Zn)”. In: *Chemical Science* 5.12 (2014), pp. 4569–4581. ISSN: 2041-6520. DOI: 10.1039/c4sc02064b.

[34] Decai Yu et al. “A combined experimental and quantum chemical study of $CO_2$ adsorption in the metal-organic framework CPO-27 with different metals”. In: *Chemical Science* 4.9 (2013), pp. 3544–3556. ISSN: 2041-6520. DOI: 10.1039/c3sc51319j.

[35] Jarad A. Mason et al. “Evaluating metal–organic frameworks for post-combustion carbon dioxide capture via temperature swing adsorption”. In: *Energy & Environmental Science* 4.8 (2011), pp. 3030–3040. ISSN: 1754-5692. DOI: 10.1039/c1ee01720a.

[36] Stephen R. Caskey, Antek G. Wong-Foy, and Adam J. Matzger. “Dramatic Tuning of Carbon Dioxide Uptake via Metal Substitution in a Coordination Polymer with Cylindrical Pores”. In: *Journal of the American Chemical Society* 130.33 (2008), pp. 10870–10871. ISSN: 0002-7863. DOI: 10.1021/ja8036096.

[37] David Britt et al. “Highly efficient separation of carbon dioxide by a metal-organic framework replete with open metal sites”. In: *Proceedings of the National Academy of Sciences* 106.49 (2009), pp. 20637–20640. ISSN: 0027-8424. DOI: 10.1073/pnas.0909718106.
